# Mathematical Modeling of Diffusion of a Hydrophilic Ionic Fertilizer in Plant Cuticles: Surfactant and Hygroscopic Effects

**DOI:** 10.3389/fpls.2018.01888

**Published:** 2018-12-20

**Authors:** E. C. Tredenick, T. W. Farrell, W. A. Forster

**Affiliations:** ^1^School of Mathematical Sciences, Queensland University of Technology, Brisbane, QLD, Australia; ^2^ARC Centre of Excellence for Mathematical and Statistical Frontiers, Queensland University of Technology, Brisbane, QLD, Australia; ^3^Plant Protection Chemistry NZ Ltd., Rotorua, New Zealand

**Keywords:** plant cuticle, ionic active ingredient, porous diffusion, adsorption, mathematical model, aqueous pores, penetration, surfactant

## Abstract

The agricultural industry requires improved efficacy of sprays being applied to crops and weeds to reduce their environmental impact and increase financial returns. One way to improve efficacy is by enhancing foliar penetration. The plant leaf cuticle is the most significant barrier to agrochemical diffusion within the leaf. The importance of a mechanistic mathematical model has been noted previously in the literature, as each penetration experiment is dictated by its specific parameters, namely plant species, environmental conditions such as relative humidity and spray formulation including adjuvant addition. A mechanistic mathematical model has been previously developed by the authors, focusing on plant cuticle diffusion of calcium chloride through tomato fruit cuticles including pore swelling, ion binding and evaporation, along with the ability to vary the active ingredient concentration and type, relative humidity and plant species. Here we further develop this model to include adjuvant effects as well as the hygroscopic nature of deliquescent ionic solutions with evaporation on the cuticle surface. These modifications to a penetration and evaporation model provide a novel addition to the literature and allow the model to be applied to many types of evaporating ionic hygroscopic solutions on many types of substrates, not just plant cuticles. We validate our theoretical model results against appropriate experimental data, discuss key sensitivities and relate theoretical predictions to physical mechanisms. The important governing mechanisms influencing surfactant enhanced penetration of ionic active through plant cuticles were found to be aqueous pore radius, pore density, cuticle thickness and initial contact angle of the applied droplet; ion binding, relative humidity and evaporation including hygroscopic water absorption parameters for point of deliquescence. The sensitivity analysis indicated surfactants increase penetration by changing the point of deliquescence of a solution, which alters the water absorption and the initial contact angle, which alters the number of pores under the droplet. The results of the validation and sensitivity analysis imply that this model accounts for many of the mechanisms governing penetration in plant cuticles.

## 1. Introduction

The agricultural industry requires improved efficacy of sprays applied to crops and weeds (Shaner and Beckie, 2014). Application by spraying is known to be effective yet often inefficient (Knoche, 1994). Improving spray efficacy can provide significant gains, including maximized crop yield and reduced: amount of active ingredient (AI) needed, spray run-off, spray volume, residues and cost (Balneaves et al., 1993; Schönherr, 2006; McKenna et al., 2013).

The plant cuticle is a dynamic structure present on the exterior of aerial plants and is considered the rate-limiting barrier to agrochemical diffusion through plant leaves (Schönherr and Riederer, 1989). It forms a protective barrier that is modified by the environment and regulates water loss (Riederer and Schreiber, 2001; Kerstiens, 2010; Yeats et al., 2012). The plant cuticle is a lamellate, porous, highly heterogeneous structure that changes in thickness, chemical composition, adaxial and abaxial cuticle surfaces, abundance and arrangement of structures such as aqueous pores between various plant species (Buchholz, 2006; Schreiber et al., 2006; Jeffree, 2008; Kerstiens, 2010). Plant species variations have a profound effect on cuticle surface structures such as trichomes, stomata, waxes and folds that affect the wettability (Koch et al., 2008) and consequently the initial contact angles of spray droplets applied to these surfaces (Nairn et al., 2013). Cuticle thickness varies significantly between species (Jeffree, 2008) and affects penetration (Schreiber and Schönherr, 2009, p. 31) of hydrophilic ionic AIs (Santier and Chamel, 1992).

Aqueous pores are dynamic structures within the cuticle that form only in the presence of water (Riederer and Schreiber, 2001; Schönherr, 2006). They are crucial for foliar penetration of agrochemicals (Schönherr, 2006). The maximum pore size changes between plant species, with estimates between 0.3 nm in *Hedera helix L*. (Popp et al., 2005) and 2.12 nm in tomato fruit cuticle membranes (Beyer et al., 2005; Schreiber and Schönherr, 2009, p. 87). These pores are classified as nanopores (Mays, 2007; Loucks et al., 2012).

Here we will focus on agrochemicals including plant hormones, growth regulators, plant nutrients [for example calcium chloride (CaCl_2_)] and pesticides such as bentazon and glyphosate that are ionic hydrophilic compounds (ionic AIs). These ionic AIs include adjuvants in the spray formulation and penetrate exclusively through aqueous pores (Schönherr and Schreiber, 2004; Schönherr, 2006) in isolated astomatous plant cuticles. Ionic AI penetration has been said to have major practical importance to the agricultural industry and significantly less is known about penetration of ionic AIs through the plant cuticle (Schreiber, 2005) but enough governing mechanisms have been defined to conduct theoretical modeling.

Ionic AIs travel through the plant cuticle via Fickian diffusion in aqueous pores (Baur, 1999; Schönherr, 2006; Schreiber and Schönherr, 2009). The concentration gradient is the driving force of diffusion (Riederer and Muller, 2008). Applying this to AI penetration in plant cuticles, an unequal distribution of ions across the cuticle creates a concentration gradient, which causes solutes to move along this concentration gradient. Water crosses the cuticle in aqueous pores via diffusion (Schreiber et al., 2006) and is also adsorbed to the aqueous pore walls as a monolayer (Luque et al., 1995; Kerstiens, 2006).

Ion binding to the cuticle surface can significantly alter penetration. When ions are bound to the cuticle surface, they are trapped and cannot travel through the cuticle. When calcium ion penetration was measured through isolated astomatous tomato fruit cuticles (*Lycopersicon esculentum*), application onto the outside surface of the cuticle produced about 3.5 times more penetration than the inside surface after 40 h (Yamada et al., 1964). A 10% difference between the penetration of glyphosate applied to the outer (highest) and inner surfaces of tomato fruit (*Lycopersicon esculentum Mill*.) cuticles at a relative humidity close to saturation after 4 days was found (Santier and Chamel, 1992). Another preliminary study found a 60% difference between the penetration of phosphorus acid (phosphite) applied onto the abaxial (highest) and adaxial whole kauri plant leaves (both were applied to the outer surfaces) (Horgan, 2017).

Adjuvants are the broad category of chemicals added to spray tank formulations to change the AIs efficacy or spray characteristics (Hazen, 2000). Adjuvants can be wetting agents, surfactants, humectants or a combination. It is important to consider the effect of adjuvant addition with ionic AI penetration. Most experiments that include some sort of adjuvant find penetration of AI is significantly increased (Schönherr, 2001; Schönherr and Luber, 2001).

Surfactants are forms of adjuvants that modify liquids surface characteristics (Hazen, 2000). They change the contact area, contact angle and surface tension of applied droplets (Gaskin et al., 2005). They improve emulsifying by keeping solutions well mixed and improve dispersing, spreading and wetting (Hazen, 2000). Surfactants are known to decrease an AI's point of deliquescence (POD), allowing further penetration to occur at lower relative humidity. Adding surfactants to a NaCl solution decreased the POD from 75%RH to 71%RH (Chen and Lee, 2001). Surfactants including vegetable oils such as ethoxylated rapeseed oil (RSO) are known to delay evaporation, alter the droplet surface tension and initial contact angle (Hazen, 2000; Haefs et al., 2002), produce a more homogeneous droplet footprint, increase the initial droplet area and subsequent droplet residue area, maintain sufficient relative humidity at the AI/cuticle interface and allow a paste-like deposit that is sufficiently wet to promote penetration over longer periods (Kraemer et al., 2009).

Relative humidity can significantly alter penetration of ionic AIs through plant cuticles. This is thought to be due to hydration of the cuticle, due to water adsorption to the aqueous pore walls (Luque et al., 1995; Kerstiens, 2006; Schönherr, 2006). High relative humidity is thought to promote pore swelling and increase the number and radius of aqueous pores, which generally leads to increased penetration (Middleton and Sanderson, 1965; Schönherr and Schmidt, 1979; Schönherr, 2000, 2002, 2006; Ramsey et al., 2005). Relative humidity also impacts the rate of spray droplet evaporation and hygroscopic water absorption (Tang et al., 1997).

Ionic compounds can absorb water in both liquid and vapor form from the air, due to their strong attractive forces for the highly polar water molecules. Calcium chloride's moisture absorption is 14 times its weight in water at 95%RH. A quantitative measure of the ability to absorb water molecules from the air, termed hygroscopicity, is given by the POD of an ionic substance. If the relative humidity is above the POD of a salt, solid crystals will absorb moisture from the air until they dissolve and remain in solution (Tang et al., 1997; Oxy Chemical Company, 2014; Absortech, 2015). The solution will continue to absorb water from the air until an equilibrium is reached between the vapor pressure of the solution and the air. Crystals of CaCl_2_ have been found to increasingly absorb water over several hours (Bouzenada et al., 2016). If the relative humidity then increases, more water will be absorbed by the solution. However, if the relative humidity then decreases, water will evaporate from the solution to the air and crystals will start to form when the relative humidity goes below the POD (Tang et al., 1997; Oxy Chemical Company, 2014). For example, the POD of CaCl_2_ is very low at 32%RH at 20°C (Kolthoff et al., 1969), so if the relative humidity is above the POD, at say 50%RH, salt crystals or salt solution will absorb water. If the relative humidity is below the POD, at say 20%RH, the solution will continue to evaporate and eventually form crystals.

Many common ionic solutions, such as sodium chloride (NaCl) and CaCl_2_, have the ability to attract water. Ionic solutions are often deposited onto mangroves (Lovelock et al., 2017), present in atmospheric particles (Burkhardt and Hunsche, 2013) and in sprinkler irrigation (Isla and Aragüés, 2009; Fernández et al., 2017). Small traces of additives such as NaCl, adjuvants, impurities and deposits on the leaf surface can significantly alter the POD of an ionic solution formulation by 4%RH to 17%RH, also changing the solutions maximum ion concentration (Chen and Lee, 2001; Fernández et al., 2017). We note the most important impact of a change in POD with an additive, applied to ionic AI penetration through plant leaves, is that more water can be absorbed, so the evaporation and penetration time is extended and hence there is more water available at the same relative humidity, increasing the area under the droplet and hence the number of aqueous pores available for penetration.

**Figure 1:**
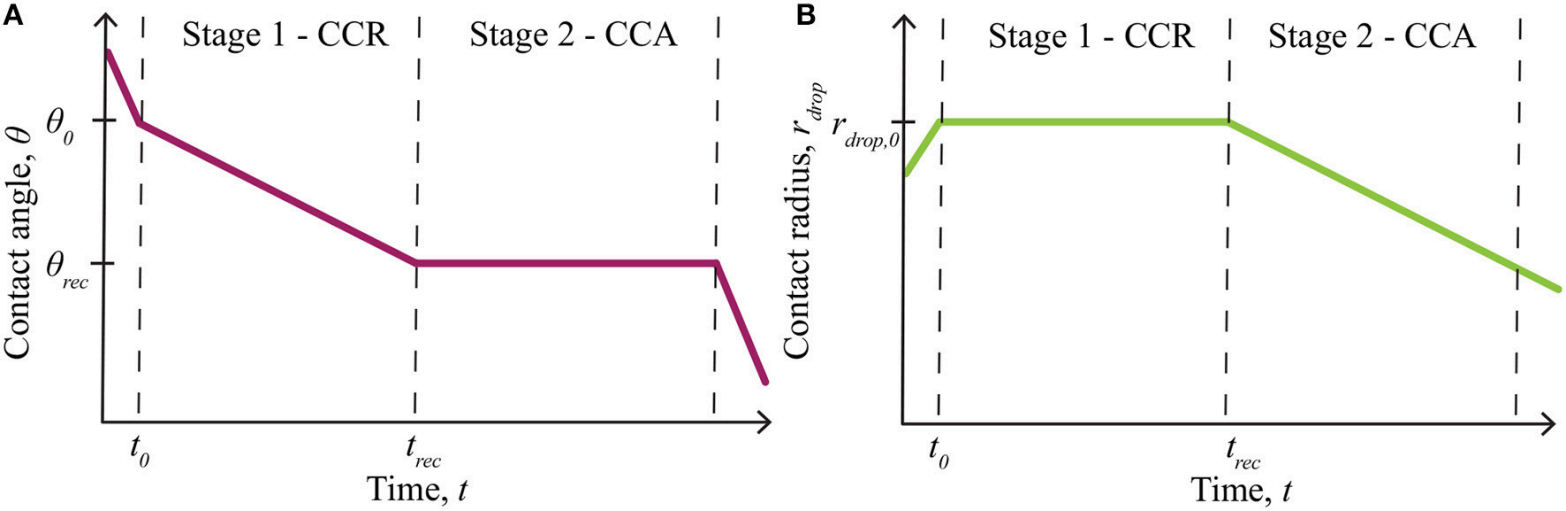
Summary of evaporation modes, including the initial spreading phase and evaporation stages for partial wetting, redrawn from Doganci et al. (2011), Semenov et al. (2013, 2014). The contact angle change is shown in **(A)** and the contact radius in **(B)**. Only stage 1 - constant contact radius (CCR) and stage 2 - constant contact angle (CCA) mode, contribute to evaporation significantly. Evaporation starts at *θ*_0_ and *r*_drop, 0_, then the contact angle, *θ*, changes in stage 1, until it reaches *θ*_rec_ at *t*_rec_. Stage 2 then begins, where the contact radius, *r*_drop_, changes from *r*_drop, 0_, until evaporation finishes. Reprinted (adapted) with permission from Semenov et al. (2013). Copyright 2013 American Chemical Society.

The effect of evaporation on AI penetration through plant cuticles is an important aspect to consider for both experimental and field work. Evaporation experiments on plant leaves are commonly performed at very low relative humidity (Zhou et al., 2017), below the POD, so do not account for hygroscopic water absorption. Theoretical evaporation models do not account for hygroscopic water absorption (Picknett and Bexon, 1977; Popov, 2005). We wish to model the deliquescent nature of ionic solutions, where the solutions can absorb water, as a function of any relative humidity and time. Evaporation can be simulated by considering a single sessile drop, deposited on a solid substrate, where the wetted area is characterized by a contact angle, contact radius and drop height, forming a spherical-cap shape (Erbil, 2012). Sessile droplet evaporation on a substrate is governed by the wettability and roughness of the substrate (Dash and Garimella, 2013), dictated by the contact radius and the contact angle of the droplet (Picknett and Bexon, 1977). These mechanisms are alternate to bulk solution evaporation (Tang et al., 1997) and a droplet suspended in air, which evaporates uniformly at a rate proportional to its radius (Dash and Garimella, 2013). The four general phases of partial wetting of an evaporating sessile drop, as shown in Figure 1 [redrawn from Doganci et al. (2011); Semenov et al. (2013, 2014)], can be described as follows (Dash and Garimella, 2013; Semenov et al., 2013, 2014):

1. Spreading: A droplet is initially deposited on a surface and quickly spreads until an initial contact angle (*θ*_0_) and radius (*r*_drop, 0_) are reached. This phase occurs in under 2 min, for adjuvant solutions on leaf surfaces (Xu et al., 2011). Evaporation can be neglected here.
2. First Stage - Constant Contact Radius Mode: The first stage of evaporation, termed constant contact radius (CCR) mode, is characterized by a changing contact angle (*θ*) and a constant contact radius (*r*_drop, 0_). The contact angle changes between the initial contact angle (*θ*_0_) and the receding contact angle (*θ*_rec_), which is found experimentally.
3. Second Stage - Constant Contact Angle Mode: The second stage of evaporation, termed constant contact angle (CCA) mode, is characterized by a constant contact angle (*θ*_rec_) and a changing contact radius (*r*_drop_).
4. Third Stage: The third and final stage of evaporation occurs over a short time and continues until the droplet completely evaporates. It is characterized by a changing contact angle and radius. This stage is usually ignored in evaporation models as it is relatively short.

In general, the first (CCR mode) and second (CCA mode) stages alone have been shown to govern evaporation, which has been confirmed both experimentally and theoretically (Semenov et al., 2013, 2014) and applies to a wide range of situations. This evaporation sequence has also been found to occur with RSO 5 (Hunsche and Noga, 2012). Dried deposit patterns of droplets of glyphosate with RSO 5 on various inorganic surfaces have been studied. Both the contact angle and radius changed during evaporation as both coffee-ring (CCR mode) and Marangoni flows (CCA mode) effects are shown along with other small island patterns. These effects change with surfactant concentration and type of surface (Hunsche and Noga, 2012).

A reliable, universally applicable, mechanistic mathematical model to simulate penetration of agrochemicals and adjuvants through plant cuticles would be beneficial as models aid to develop understanding of the governing mechanisms. Penetration experimental results are specific to each AI, formulation including adjuvant, plant species combination and environmental conditions including relative humidity. It has been noted that progress of agrochemical efficacy will not be made until appropriate models are created to simulate the multiple complex processes involved (Zabkiewicz, 2007). An interdisciplinary approach is needed in order to study these complex mechanisms.

Current mathematical models of penetration of ionic AIs have been created as alternatives to time consuming and expensive field/laboratory studies and fall into two categories: the first employ empirical expressions that require a rate constant to be measured specific to each different plant species, hydrophilic AI, adjuvant and environmental conditions (Forster et al., 2006; Schreiber and Schönherr, 2009, p. 132) and the second incorporate diffusion (Hsu, 1983; Satchivi et al., 2000; Veraverbeke et al., 2003; Mercer, 2007; Pecha et al., 2012). Comprehensive reviews can be found elsewhere (Forster et al., 2004; Trapp, 2004; Tredenick et al., 2017). These models, however, do not include important governing mechanisms such as ion binding, sessile droplet evaporation with water absorption including POD and relative humidity. The authors previously introduced a mechanistic mathematical model in Tredenick et al. (2017) to simulate ionic agrochemical penetration through plant cuticles with sessile droplet evaporation, the ability to vary plant species, type and initial concentration of active ingredient, relative humidity and ion binding. Adjuvants were not included and only a simplistic evaporation formulation was used, which did not consider the POD or water absorption of ionic AI solutions. Here we wish to extend this model to include these mechanisms.

We aim to simulate the complex mechanisms involved in adjuvant enhanced hydrophilic ionic AI penetration through plant cuticles using a predictive mathematical model. This model will account for relative humidity, plant species variations and adjuvant effects. Specifically, we incorporate new mechanisms of water absorption, POD with evaporating droplets and effects of adding adjuvants such as the surfactant RSO 5. We seek to validate the predictions of our model against well-established experimental data and discuss the key implications of the models' sensitivities.

## 2. Model Framework

The formulation of a mathematical model is based on an understanding of the experimental setup used. Here we will verify the solution of the model against well-established data from the experimental setup described in Kraemer et al. (2009), who studied the penetration of CaCl_2_ and RSO 5 from droplets, applied to astomatous, isolated tomato fruit (*Solanum lycopersicum* L., cultivar “Panovy”) cuticles. Droplets with known concentrations were applied to the adaxial cuticle surface. A receiver solution in a water bath was placed in close contact with the inner surface of the cuticle, which was analyzed at regular intervals for CaCl_2_. Figure S1 shows the Kraemer et al. (2009) data for both CaCl_2_ (A) and CaCl_2_ with RSO 5 (B). We have presented their data as a mass percentage of the initial applied CaCl_2_, to further understand their results. The optimal way to visualize penetration results is as a mass percentage. We have fitted their data with a linear combination of exponential functions. Refer to the Supplementary Materials for further details. Two exponential terms were fitted to the data, which indicates there are at least two separate timescales involved in this penetration, governing at least two different mechanisms. In Figure S1A for CaCl_2_, the most important timescale is the first 5 h and penetration after this time occurs at a much slower rate. This rapid then slow penetration is well documented for ionic penetration through plant cuticles (Schönherr, 2006). Figure S1A shows that the mean percentage penetration for CaCl_2_ is 40% at 48 h and penetration is not very sensitive to the initial concentration of AI (when the outlier 25 *μ*g is excluded). This suggests that around 60% of AI has not been taken up through the cuticle. In Figure S1B for CaCl_2_ with RSO 5, penetration is much higher than without RSO 5 (the mean penetration is 56.2% compared to 40% at 48 h). The mean percentage penetration was found to be 56.2% with a standard deviation of 8.4% at 48 h, hence the final penetration at 48 h is very similar across all initial concentrations. The penetration initially occurs rapidly, then levels off after 20 min.

Kraemer et al. (2009) have provided data for the initial droplet area, shown in Table 1 for CaCl_2_ and CaCl_2_ with RSO 5. With spherical cap geometry, we can numerically approximate the initial contact angle, *θ*_0_, from the known initial droplet area and volume, the equation for *A*_drop,0_ in Table 2, using the secant method (Wolfe, 1959). These values vary significantly with initial CaCl_2_ concentration, with a nonlinear trend. The angle where CCA mode begins, termed the receding contact angle, *θ*_rec_, is difficult to measure experimentally (Semenov et al., 2014). However, *θ*_rec_ can be estimated based on the behavior of experimental data with comparable initial contact angles (Stauber et al., 2015) and we find the contact angle should decrease by approximately 12.64%. Therefore, we reduce the values of *θ*_0_ in Table 1 by 12.64% to find *θ*_rec_.

**Table 1:** Initial droplet area from Kraemer et al. (2009), with varying CaCl_2_ concentrations and RSO 5 addition.

**Surfactant**	**Initial CaCl_2_** **conc. (g/L)**	**Initial droplet** **area (m^**2**^)**	***θ*_0_** **calculated**	***θ*_rec_** **calculated**
None	1	0.49e−6	141.00	123.18
	5	0.84e−6	126.58	110.58
	10	0.86e−6	125.82	109.91
	15	0.57e−6	137.50	120.12
	30	0.55e−6	138.36	120.87
RSO 5	1	1.99e−6	87.91	76.79
	5	2.15e−6	83.32	72.79
	10	2.15e−6	83.32	72.79
	15	2.10e−6	84.73	74.02
	30	2.18e−6	82.48	72.06

**Table 2:** Model parameters.

**Parameter**	**Definition**	**Value and units**	**Comments**
*A*_Π_	Control volume area	m^2^	
*A*_drop_(*t*)	Drop surface contact area	m^2^	Surface contact area of drop on cuticle surface, Erbil et al., 2002
*A*_drop, 0_	Initial drop surface contact area on cuticle surface	m^2^	$A_{\text{drop},0}=\pi^{\frac{1}{3}}{\left(3\;g\left(\theta_{0}\right)\;V_{0}\right)}^{\frac{2}{3}}$ Erbil et al., 2002
AI	Active ingredient		
*b*	Thickness of cuticle	1.87e−5 m	Chamel et al., 1991
$c_{\text{AI},0}^{\text{drop}}$	Concentration of AI in drop at *t* = 0	mol/m^3^	Kraemer et al., 2009
*c*_mass%_	Solution weight percentage of CaCl_2_	%	Refer to discussion around Figure 2A
$c_{\text{H}{}_{2}\text{O}}^{\text{pure}}$	Pure water concentration at 20°C and *t* = 0	55,409.78 mol/m^3^	calculated
*c*_POD_	POD concentration	mol/m^3^	Refer to discussion around Equation (37)
CCR	Constant contact radius evaporation mode		
CCA	Constant contact angle evaporation mode		
*c*_*i*_(*x, t*)	Concentration of component *i* in plant cuticle	mol/m^3^	
$D_{\text{AI}}^{\text{bulk}}$	Self/bulk diffusion coefficient of AI	7.93e−10 m^2^/s	For CaCl_2_, Ca^2+^ diffuses the slowest, so Ca^2+^ value is used, Yuan-Hui and Gregory, 1974
$D_{\text{H}{}_{2}\text{O}}^{\text{bulk}}$	Self/bulk diffusion coefficient of water	2.299e−9 m^2^/s	Holz et al., 2000
*D*_evap_	Diffusivity of water in air	2.4e−5 m^2^/s	Semenov et al., 2013
*D*_*i*_(*x, t*)	Diffusivity of component *i*	m^2^/s	Liu and Nie, 2001
*F*_s_	Fractal scaling dimension	1.203 (-)	1 < *F*_*s*_ < 2 (fitted)
*f*(*θ*)	Functional variation of *θ*		Popov, 2005
*g*(*θ*)	Functional of *θ*		Popov, 2005
*H*	Relative humidity	0.7 (70%)	Kraemer et al., 2009
*i*	Component AI (CaCl_2_) or H_2O		
*k*	Ion binding reaction rate constant	8.68e−16 m^3^/s	(fitted)
*L*	Control volume length	1 m	
*M*_w, H_2_*O*_	Molecular weight H_2*O*_	18.015 g/mol	
*M*_w, AI_	Molecular weight of CaCl_2_	110.98 g/mol	
*m*_∞_	Equilibrium mass of water absorbed per CaCl_2_ applied	g_H_2_*O*_/g_AI_	Refer discussion around Figure 2A
*N*_A_	Avogadro constant	6.02214e23 mol^−1^	
*n*_0_	Number of aqueous pores on 1 m^2^ of cuticle	(-)	Equals value of η_pore_
*P*_v_	Saturated water vapor pressure in air at 20°C	2338.8 Pa	Lide, 2004
POD	Point of deliquescence		32%RH for CaCl_2_ Kolthoff et al., 1969 and 27%RH for CaCl_2_ with RSO 5
R	Gas constant	8.3145 Pa·m^3^/K/mol	
RSO	Rapeseed oil surfactant		
*r*_drop_	Drop contact radius	m	Contact radius of drop on cuticle surface
*r*_drop, 0_	Initial contact radius of drop on cuticle surface	m	$r_{\text{drop,0}}={\left(3\;g\left(\theta_{0}\right)\;V_{0}/\pi \right)}^{\frac{1}{3}}$ Erbil et al., 2002
*r*_H_2_*O*_	Van der Waals radius of a water molecule	1.5e−10 m	Schreiber et al., 2006
*r*_p_(*x, t*)	Radius of aqueous pore	m	
$r_{\text{p}}^{\text{max}}$	Maximum radius of aqueous pores	2.12e−9 m	For tomato fruit cuticle, (Schreiber and Schönherr, 2009, p. 87)
*RH*	Relative humidity in text		
*t*	Time	s	
*T*	Temperature	293.15 K (20°C)	Kraemer et al., 2009
*u*	dummy or bound integration variable		
*V*_0_	Volume of droplet at *t* = 0	1e−9 m^3^	Kraemer et al., 2009
$V_{\text{H}{}_{2}O}^{\text{drop}}\left(t\right)$	Volume of water in droplet at time *t*		
*V*_Del_(*t*)	Deliquescent droplet volume	m^3^	
${\bar{v}}_{\text{AI}}$	Partial molar volume CaCl_2_	1.6e−5 m^3^/mol	Oakes et al., 1995
${\bar{v}}_{\text{H}{}_{2}O}$	Partial molar volume water	1.8047e−5 m^3^/mol	Zen, 1957
*x*	Length	m	
β_H_2_*O*_	Langmuir parameter	3.77e−5 m^3^/mol	Equilibrium parameter of adsorbed water
ε(*x, t*)	Porosity of cuticle	(-)	0 < ε < 1
η_pore_	Density of aqueous pores in cuticle	2.18e15 m^−2^	(fitted)
γ	Reduction factor for initial pore size	0.97	
Γ_H_2_*O*_(*x, t*)	Concentration of water adsorbed per unit area at equilibrium	mol/m^2^	Luque et al., 1995; Bard and Faulkner, 2001
Γ_S_	Langmuir saturation constant	9.6832e−4 mol/m^2^	0 < Γ_H_2_*O*_ < Γ_S_, saturation concentration of water adsorbed in aqueous pores per unit area
Λ	Evaporation constants as a function of relative humidity	m^2^/s	Λ = *D*_evap_ ψ/ρ_H_2_*O*_ Erbil, 2012
ψ(*H*)	Saturated water vapor concentration as a function of relative humidity	g/m^3^	ψ = *M*_w, H_2_*O*_ *P*_v_(1−*H*)/(*R T*) Erbil, 2012
Φ	Shifting factor for *m*_∞_ and *c*_mass%_		
ρ_H_2_*O*_	Liquid density H_2_O at 20°C	9.98207e5 g/m^3^	Weast and Lide, 1989
ρ_AI_	Liquid density of CaCl_2_ at 20°C	2.16e6 g/m^3^	Dow Chemical Company, 2003
*θ*(*t*)	Contact angle of drop on cuticle surface that changes with time	rads	
*θ*_0_	Contact angle of drop on cuticle surface at *t* = 0	rads	For CaCl_2_ with RSO5, refer to Table 1
*θ*_rec_	Receding contact angle of drop on cuticle surface	rads	Refer to Table 1
χ	Logistic decay evaporation constant	0.043 L^2^/g^2^	(fitted)
χ	Logistic decay evaporation term (a constant) as a function of initial concentration of AI		calculated
ξ	POD shifting factor to incorporate with the addition of adjuvants	5%RH	

*Dimensionless parameters are shown in the Supplementary Materials*.

**Figure 2:**
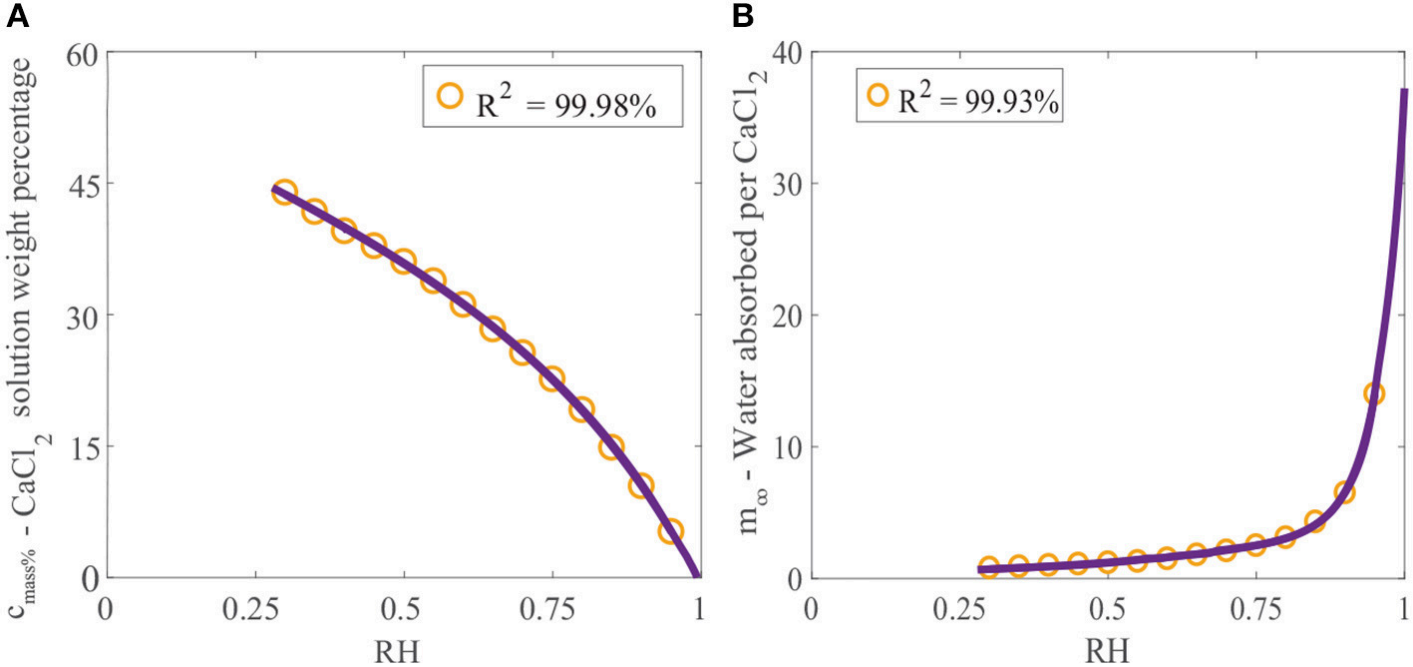
**(A)** Effect of relative humidity on final solution weight percentage of CaCl_2_ and **(B)** effect of relative humidity on water absorption over CaCl_2_ (Dow Chemical Company, 2003). We have fitted the data, shown as the orange circles, with Equation (1) for *c*_mass%_, and Equation (2) for *m*_∞_, shown as the purple line.

Crystallization of a solution of CaCl_2_ will not occur at 20 °C if the relative humidity is above the POD as the solution will absorb water due to its hygroscopic nature (Dow Chemical Company, 2003). If the relative humidity is below the POD, evaporation will continue and the solution will eventually form crystals. Data published for the final solution weight percentage of CaCl_2_ can be used to find the maximum concentration that CaCl_2_ can reach before crystallization occurs as a function of relative humidity (Dow Chemical Company, 2003). This data can be fitted, as shown in Figure 2A, using the linear combination of two exponential functions:

(1) $$\begin{array}{ll} c_{\text{mass}\%}\left(\Phi \right) & =-0.8307\;{\text{e}}^{3.618\;\Phi }+55.44\;{\text{e}}^{-0.612\;\Phi }, \end{array}$$

where *c*_mass%_ is the final solution weight percentage of CaCl_2_ as a function of the relative humidity adjustment factor, Φ. The advantage of fitting this data is that any relative humidity can be utilized.

The effect of relative humidity on water absorption over CaCl_2_ at 25°C has been studied (Dow Chemical Company, 2003). Data for the mass of water absorbed per mass of CaCl_2_ applied (g_H_2_O_/g_CaCl_2__) with relative humidity has been provided. Again, this data can be fitted using a linear combination of two exponential functions:

(2) $$\begin{array}{ll} m_{\infty }\left(\Phi \right) & =0.307\;{\text{e}}^{2.763\;\Phi }+1.218\times 10^{-9}\;{\text{e}}^{24\;\Phi }, \end{array}$$

where *m*_∞_ is the equilibrium or maximum of the ratio of the mass of water absorbed per mass of CaCl_2_ applied (g_H_2_O_/g_CaCl_2__). Figure 2B shows the data fitted with Equation (2). The relative humidity has a large effect on water absorption over CaCl_2_. In Figure 2B we can see that at high relative humidities, CaCl_2_ absorbs very large volumes of water. At 95%RH, CaCl_2_ can absorb 14 times its mass in water.

The POD of an ionic solution can change with the addition of an adjuvant (Chen and Lee, 2001; Fernández et al., 2017). If the POD of an ionic solution changes, the water absorption over the solution changes. As the POD of CaCl_2_ with RSO 5 is unknown, we will assume RSO 5 changes the POD of CaCl_2_ by 5%RH, similar to that found by Chen and Lee (2001). This changes the POD of CaCl_2_ from 32%RH (Kolthoff et al., 1969) to 27%RH with RSO 5. The 5%RH change to the POD is referred to as ξ, and is a parameter that is easily altered within the model. When further research has been carried out in the future, this parameter can be modified.

With no surfactant, a relative humidity of 70%RH (Kraemer et al., 2009) is used to find *c*_mass%_ and *m*_∞_. Fernández et al. (2017) found when the POD changed, so did the maximum solution concentration, *c*_mass%_. To find *c*_mass%_ and *m*_∞_, in Equations (1–2), with the new POD, we assume a one-to-one relationship. When the POD is shifted with the addition of RSO 5, the value used to find *c*_mass%_ and *m*_∞_, or the independent variable in Equations (1–2), Φ, is shifted from 70%RH to 75%RH.

If the POD is greater or equal to the relative humidity, *c*_mass%_ and *m*_∞_ are not included in the evaporation formulation. If the ionic AI or surfactant changes, producing a new POD, a new data set can be found experimentally, for *c*_mass%_ and *m*_∞_, which can be fitted with a new function for *c*_mass%_ and *m*_∞_.

### 2.1. Model Development

**Figure 3:**
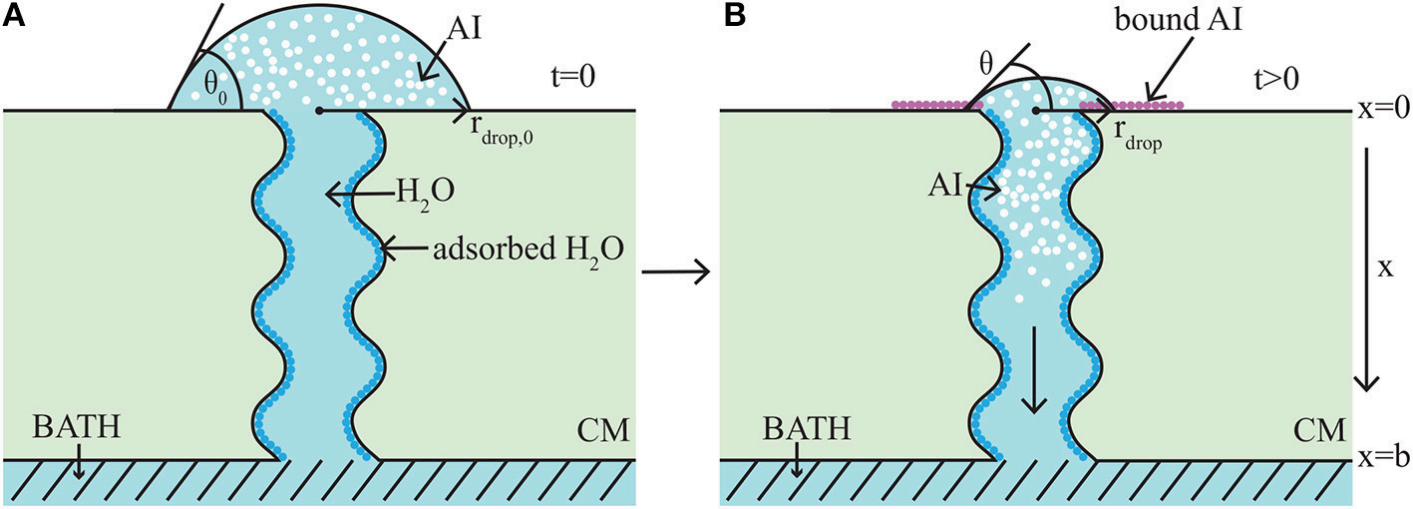
1-D cuticle model domain of AI diffusion and water adsorption-diffusion. The initial conditions of the model are shown on the left **(A)** and a short time later on the right **(B)**. Diffusion of AI starts at the outer surface (at *x* = 0), where a drop of solution containing AI and water having initial contact angle, *θ*_0_, and radius, *r*_drop, 0_, sits. Over time AI travels through the porous cuticle membrane (CM) to the well stirred water bath at the inner surface (at *x* = *b*). Water adsorbs to the surface of the pore (shown as dark blue circles). In **(B)** at the cuticle surface (at *x* = 0), AI is trapped on the surface via ion binding, (shown as pink circles) and the droplet solution has undergone some evaporation and both the contact angle, *θ*, and radius, *r*_drop_, have changed. For simplicity, a single tortuous aqueous pore can be seen crossing the cuticle (not to scale). Figure adapted from a figure published in Tredenick et al. (2017). The article was published under a Creative Commons CC-BY license.

The model takes the form of a nonlinear, quasi-one-dimensional, diffusion model. We will briefly describe the modeling formulation, however we note that a full description can be found in the authors previous work (Tredenick et al., 2017). A schematic diagram of the model domain based on the experimental setup (Kraemer et al., 2009) is shown in Figure 3. Both AI and water diffuse and all variables change primarily along the cuticle membranes thickness, *x* (0 ≤ *x* ≤ *b*). In Figure 3, the initial condition is shown on the left (A) and a short time later on the right (B). In Figure 3A, initially a droplet with known initial contact angle, *θ*_0_, radius, *r*_drop, 0_, concentration of AI (CaCl_2_), $c_{\text{AI,0}}^{\text{drop}}$ and RSO 5 is placed on the outer cuticle surface, at *x* = 0. A well stirred water bath exists at the inner surface, at *x* = *b*. A single (for clarity) tortuous aqueous pore can be seen traversing the cuticle. In Figure 3B, after a short time later, some AI has diffused from the droplet into the cuticle toward the well stirred water bath. The droplet changes in size due to evaporation, hygroscopic effects and diffusion. Both the radius, *r*_drop_, and contact angle, *θ*, of the droplet have changed. Ions of AI lost to ion binding can be seen as pink circles attached to the outer cuticle surface, which cannot diffuse. Adsorbed water is seen attached to the aqueous pore walls, shown as dark blue circles.

The model, including the variables and parameters as described in Table 2 along with the governing partial differential equations, initial conditions (ICs), boundary conditions (BCs) and auxiliary functions, which is similar to previous work by the authors (Tredenick et al., 2017), is as follows:

(3) $$\begin{array}{lll} \frac{\partial \left(\varepsilon c_{\text{AI}}\right)}{\partial t} & = & -\frac{\partial }{\partial x}\left[-D_{\text{AI}}\frac{\partial \left(\varepsilon c_{\text{AI}}\right)}{\partial x}\right],\;0<x<b,\;t>0, \end{array}$$

(4) $$\begin{array}{l} \frac{\partial (\varepsilon c_{{\text{H}}_{\text{2}}\text{O}})}{\partial t}=-\frac{\partial }{\partial x}\left[-D_{{\text{H}}_{\text{2}}\text{O}}\frac{\partial (\varepsilon c_{{\text{H}}_{\text{2}}\text{O}})}{\partial x}\right]\\ \;-\frac{2}{r_{\text{p}}}(1-\varepsilon )\frac{\partial \Gamma_{{\text{H}}_{\text{2}}\text{O}}}{\partial t},\;0<x<b,\;t>0, \end{array}$$

(5) $$\begin{array}{l} \text{Functions}:\;r_{\text{p}}\left(x,t\right)=r_{\text{H}{}_{2}\text{O}}\left[1+{\left(\sin\left({\left(\Gamma_{\text{H}{}_{2}\text{O}}\;r_{\text{H}{}_{2}\text{O}}^{2}\;N_{\text{A}}\right)}^{-1}\right)\right)}^{-1}\right],\\ \;0<x<b,\;t>0, \end{array}$$

(6) $$\begin{array}{ll} \varepsilon \left(x,t\right)=\pi {\left[\frac{r_{\text{p}}}{L}\left(\sqrt{n_{0}}+1\right)\right]}^{2}, & \;0<x<b,\;t>0, \end{array}$$

(7) $$\begin{array}{ll} D_{\text{AI}}\left(x,t\right)=D_{\text{AI}}^{\text{bulk}}\;\varepsilon^{\left(\frac{F_{\text{s}}}{2-F_{\text{s}}}\right)}, & \;0<x<b,\;t>0, \end{array}$$

(8) $$\begin{array}{ll} D_{\text{H}{}_{2}\text{O}}\left(x,t\right)=D_{\text{H}{}_{2}\text{O}}^{\text{bulk}}\;\varepsilon^{\left(\frac{F_{\text{s}}}{2-F_{\text{s}}}\right)}, & \;0<x<b,\;t>0, \end{array}$$

(9) $$\begin{array}{ll} \Gamma_{\text{H}{}_{2}\text{O}}\left(x,t\right)=\frac{\Gamma_{\text{S}}\;\beta_{\text{H}{}_{2}\text{O}}\;c_{\text{H}{}_{2}\text{O}}}{1+\beta_{\text{H}{}_{2}\text{O}}\;c_{\text{H}{}_{2}\text{O}}}, & \;0<x<b,\;t>0, \end{array}$$

(10) $$\begin{array}{ll} \text{ICs}:\;c_{\text{AI}}\left(x,0\right)=0, & \;0<x<b, \end{array}$$

(11) $$\begin{array}{ll} c_{\text{AI}}\left(0,0\right) & =c_{\text{AI,0}}^{\text{drop}}, \end{array}$$

(12) $$\begin{array}{ll} r_{\text{p}}\left(x,0\right) & =r_{\text{p}}^{\text{max}}\;\gamma ,\;0\leq x\leq b \end{array}$$

(13) $$\begin{array}{ll} c_{\text{H}{}_{2}\text{O}}\left(x,0\right) & =c_{\text{H}{}_{2}\text{O}}^{\text{pure}},\;0<x<b, \end{array}$$

(14) $$\begin{array}{ll} c_{\text{H}{}_{2}\text{O}}\left(0,0\right) & =\frac{1-{\bar{v}}_{\text{AI}}c_{\text{AI}}\left(0,0\right)}{{\bar{v}}_{\text{H}{}_{2}\text{O}}}, \end{array}$$

(15) $$\begin{array}{l} \Gamma_{\text{H}{}_{2}\text{O}}\left(x,0\right)={\left(r_{\text{H}{}_{2}\text{O}}^{2}\;N_{A}\arcsin\left({\left(\frac{r_{\text{p}}\left(x,0\right)}{r_{\text{H}{}_{2}\text{O}}}-1\right)}^{-1}\right)\right)}^{-1},\\ \;0<x<b, \end{array}$$

(16) $$\begin{array}{ll} \beta_{\text{H}{}_{2}\text{O}} & ={\left(c_{\text{H}{}_{2}\text{O}}\left(x,0\right)\left[\frac{\Gamma_{\text{S}}}{\Gamma_{\text{H}{}_{2}\text{O}}\left(x,0\right)}-1\right]\right)}^{-1},\;0<x<b, \end{array}$$

(17) $$\begin{array}{lll} \text{BC - AI (bath)}:\;c_{\text{AI}}\left(b,t\right) & =0, & t>0, \end{array}$$

(18) $$\begin{array}{lll} \text{BC - H}{}_{2}\text{O (drop)}:\;c_{\text{H}{}_{2}\text{O}}\left(0,t\right) & =\frac{1-{\bar{v}}_{\text{AI}}c_{\text{AI}}\left(0,t\right)}{{\bar{v}}_{\text{H}{}_{2}\text{O}}},\; & t>0, \end{array}$$

(19) $$\begin{array}{lll} \text{BC - H}{}_{2}\text{O (bath)}:\;c_{\text{H}{}_{2}\text{O}}\left(b,t\right) & =c_{\text{H}{}_{2}\text{O}}^{\text{pure}}, & t>0. \end{array}$$

The AI can travel through plant cuticle aqueous pores via Fickian diffusion (Equation 3), which depends on the swelling of aqueous pores due to adsorbed water molecules (Kerstiens, 2006; Schreiber and Schönherr, 2009). Water facilitates AI diffusion (Equation 4), by diffusing through aqueous pores first and opening up the pores by adsorption (Kerstiens, 2006). Both transport and reaction are nonlinear. Porosity limits diffusion through the cuticle and is based on the changing aqueous pore radius. It is necessary to model porosity as aqueous pores are known to change in size. Pores are assumed to be evenly distributed on the cuticle surface with circular cross sections. The number of pores on the area *L*^2^ is given by $n_{0}=\eta_{\text{pore}}\;L^{2}$. The model accounts for tortuous pores with diffusivities for AI and water that depend on both porosity and tortuosity described by the fractal scaling dimension (Equations 7–8) (Liu and Nie, 2001; Yuan and Sundén, 2014). The concentration of water molecules per unit area adsorbed as a monolayer to the aqueous pore walls is determined with a Langmuir isotherm (Equation 10) (Giles et al., 1974; Luque et al., 1995; Bard and Faulkner, 2001).

### 2.2. Initial Conditions

The initial conditions of the model are given in Equations (10–16). Equation (10) states there is no AI in the cuticle aqueous pores initially. Equation (11) states that there is initially a constant concentration of AI applied in the droplet solution, $c_{\text{AI,0}}^{\text{drop}}$. The initial condition in Kraemer et al. (2009) for the aqueous pore size is somewhere between partially and fully swelled due to cuticle rehydration (author communication). We will assume the aqueous pores are initially at their maximum size, $r_{\text{p}}^{\text{max}}$. Equation (14) is simply Equation (18) at *t* = 0. The initial values for Γ_H_2_O_ and β_H_2_O_ in Equations (15) and (16) are found by rearranging Equations (5) and (9), and Γ_S_ can be found by assuming the pore radius is at its maximum when the pore surface is fully saturated and substituting $r_{\text{p}}=r_{\text{p}}^{\text{max}}$ and Γ_H_2_O_ = Γ_S_ into Equation (15).

### 2.3. Boundary Conditions

The BC for the concentration of AI at the bath is shown in Equation (19). The water bath is well stirred, so *c*_AI_ is zero. Equation (20) is a conservation of volume statement for the concentration of water in the droplet, *c*_H_2_O,_, based on the concentration of AI in the drop and the partial volume of AI and water, ${\bar{v}}_{\text{AI}}$ and ${\bar{v}}_{{\text{H}}_{2}\text{O}}$. The mechanisms involved in penetration at the surface of the cuticle are important, so effects such as evaporation due to environmental conditions, hygroscopic water absorption and ion binding are incorporated. Only water evaporates from the drop. The AI concentration on the outer cuticle surface in the droplet is governed by:

(20) $$\begin{array}{l} \text{ BC - AI}\left(\text{drop}\right):\;\frac{\text{d}}{\text{d}t}\left(V_{{\text{H}}_{2}\text{O}}^{\text{drop}}(t)\;c_{\text{AI}}\left(0,t\right)\right)=-k\;c_{\text{AI}}\left(0,t\right)\\ +\;\eta_{\text{pore}}A_{\text{drop}}\left(t\right)\;A_{\Pi }{\left[D_{\text{AI}}\left(x,t\right)\frac{\partial }{\partial x}\left(\varepsilon \left(x,t\right)c_{\text{AI}}\left(x,t\right)\right)\right]|}_{x=0}. \end{array}$$

As water evaporates from the drop the concentration of AI in the drop, *c*_AI_, increases (left hand side of Equation 23), then as the AI is transported from the drop into the cuticle (right hand side of equation) the concentration of AI in the drop decreases, governed by the circular cross sectional area of the control volume cylinder, *A*_Π_, diffusivity of AI, *D*_AI_, number of pores under the drop, η_pore_ *A*_drop_, and porosity of the cuticle, ε. Ions are bound to the cuticle surface and trapped (Yamada et al., 1964), incorporated into Equation (20) using a reaction rate constant, *k*. If *k* is a non-zero number, the total percentage penetration of AI cannot reach 100%.

To account for the evaporation of a sessile droplet of an ionic AI and surfactant on a plant cuticle surface, we will assume evaporation occurs as the sequence of CCR mode followed by CCA mode. We will utilize the model described in Popov (2005) and expanded upon in Dash and Garimella (2013). The Popov (2005) quasi-steady sessile droplet evaporation model is an analytical diffusion model. It is valid over the entire range of contact angles (0°−150°), and can be applied to superhydrophobic substrates. The initial droplet contact angle varies significantly between different plant species (Prüm et al., 2011; Delele et al., 2016). Therefore, the model can be applied to many physical situations such as the large variations in plant cuticle surfaces causing a wide range of initial contact angles. The Popov (2005) formulation assumes the droplet is sufficiently small so gravitational effects can be neglected. Zhou et al. (2017) have validated the Popov (2005) model with surfactant solutions on rice leaves. Evaporation is first governed by CCR mode, where the contact angle, *θ*, changes and the radius remains constant, *r*_drop, 0_. CCR mode (Popov, 2005) is characterized by:

(21) $$\begin{array}{lll} \text{CCR mode}:\;\frac{d\theta }{dt} & =\frac{-\Lambda \;{\left(1+\cos\left(\theta \right)\right)}^{2}\;f\left(\theta \right)}{r_{\text{drop,0}}^{2}}, & 0<t\leq t_{\text{rec}}, \end{array}$$

(22) $$\begin{array}{lll} \frac{dV_{{\text{H}}_{2}O}^{\text{drop}}}{dt} & =-\pi \;\Lambda \;r_{\text{drop,0}}\;f\left(\theta \right), & 0<t\leq t_{\text{rec}}. \end{array}$$

To utilize the Popov (2005) model, the following auxiliary equations based on spherical-cap geometry (Picknett and Bexon, 1977; Erbil, 2012) are included:

(23) $$\begin{array}{lll} g\left(\theta \right) & = & \frac{\sin^{3}\left(\theta \right)}{{\left(1-\cos\left(\theta \right)\right)}^{2}\;\left(2+\cos\left(\theta \right)\right)}, \end{array}$$

(24) $$\begin{array}{lll} r_{\text{drop}}\left(t\right) & = & {\left(\frac{3\;g\left(\theta \right)\;V_{{\text{H}}_{2}O}^{\text{drop}}\left(t\right)}{\pi }\right)}^{\frac{1}{3}}, \end{array}$$

(25) $$\begin{array}{ll} A_{\text{drop}}\left(t\right)= & \pi^{\frac{1}{3}}{\left(3\;g\left(\theta \right)\;V_{{\text{H}}_{2}O}^{\text{drop}}\left(t\right)\right)}^{\frac{2}{3}},\\ \text{ where }\theta \left(t\right). \end{array}$$

The functional variation of the contact angle, *f*(θ), and the receding time, *t*_rec_, which can be formulated by integrating Equation (21), are as follows (Popov, 2005):

(26) $$\begin{array}{l} f\left(\theta \right)=\tan\left(\frac{\theta }{2}\right)+8\overset{\infty }{\underset{0}{\int }}\cosh^{2}\left(\theta u\right)\;\text{csch}\left(2\pi u\right)\;\tanh\left[\left(\pi -\theta \right)u\right]\;du. \end{array}$$

(27) $$\begin{array}{l} t_{\text{rec}}=\overset{\theta_{0}}{\underset{\theta_{\text{rec}}}{\int }}\frac{r_{\text{drop,0}}^{2}}{\Lambda \;{\left(1+\cos\left(\theta \right)\right)}^{2}\;f\left(\theta \right)}\;d\theta . \end{array}$$

If the relative humidity is above the POD, hygroscopic droplet growth will occur, therefore this effect needs to be included in a mechanistic model. A novel approach to a mechanistic evaporation model is to incorporate water absorption due to the hygroscopic nature of ionic AI solutions. We have formulated the following for CCA mode, where the radius, *r*_drop_, changes and the contact angle is constant at *θ*_rec_:

(28) $$\begin{array}{l} \text{CCA mode}:\;\frac{dV_{{\text{H}}_{2}O}^{\text{drop}}}{dt}=-\pi \;\Lambda \;f\left(\theta_{\text{rec}}\right){\left(\frac{3\;g\left(\theta_{\text{rec}}\right)\;V_{{\text{H}}_{2}O}^{\text{drop}}\left(t\right)}{\pi }\right)}^{\frac{1}{3}}\\ \times \left[\frac{\chi }{V_{0}}\;V_{{\text{H}}_{2}O}^{\text{drop}}\left(t\right)\;\left(\frac{V_{{\text{H}}_{2}O}^{\text{drop}}\left(t\right)}{V_{\text{Del}}\left(t\right)}-1\right)\right]\;\left(1-\frac{c_{\text{AI}}\left(0,t\right)}{c_{\text{POD}}}\right)\\ +\frac{\eta_{\text{pore}}\;A_{\Pi }\;M_{\text{w},{\text{H}}_{2}O}\;A_{\text{drop}}\left(t\right)}{\rho_{{\text{H}}_{2}O}}\left[D_{{\text{H}}_{2}O}\left(x,t\right)\frac{\partial }{\partial x}\left(\varepsilon \left(x,t\right)c_{{\text{H}}_{2}O}\left(x,t\right)\right)\right]{|}_{x=0},\\ H>\text{POD},\;t>t_{\text{rec}}. \end{array}$$

The first line of Equation (28) is derived in Popov (2005). The second and third lines of Equation (28) are explained as follows. The droplet volume, $V_{{\text{H}}_{2}O}^{\text{drop}}$, approaches the deliquescent droplet volume, *V*_Del_. This volume, *V*_Del_, is the minimum that the volume of water in the droplet, $V_{{\text{H}}_{2}O}^{\text{drop}}\left(t\right)$, can reach at a given point in time, POD and relative humidity. When the droplet volume, $V_{{\text{H}}_{2}O}^{\text{drop}}$, approaches *V*_Del_, the evaporation rate tends to zero. This can be achieved with the addition of a logistic decay model. In Equation (28) this is achieved via the addition of a logistic decay term, namely,

$$\begin{array}{l} \frac{\chi }{V_{0}}\;V_{{\text{H}}_{2}O}^{\text{drop}}\left(t\right)\;\left(\frac{V_{{\text{H}}_{2}O}^{\text{drop}}\left(t\right)}{V_{\text{Del}}\left(t\right)}-1\right), \end{array}$$

where χ is a positive constant for logistic decay and describes the rate of maximum evaporation decrease and *V*_0_ is the initial droplet volume, $V_{{\text{H}}_{2}O}^{\text{drop}}$. At late times, $V_{{\text{H}}_{2}O}^{\text{drop}}$ approaches *V*_Del_, but does not reach it due to the asymptotic nature of the logistic term. To prevent the concentration of AI going beyond *c*_POD_, we multiply the evaporation formulation in Equation (34) by the term (1−*c*_AI_/*c*_POD_). The logistic decay evaporation constant that multiplies the evaporation in Equation (34) is described as follows:

(29) $$\begin{array}{l} \chi =\chi \;c_{\text{AI,0}}^{\text{drop}}{\;}^{2}, \end{array}$$

where χ is the logistic decay evaporation term (a constant), χ is the logistic decay evaporation constant and $c_{\text{AI,0}}^{\text{drop}}$ is the initial concentration of AI in the drop in g/L. The parameter χ in Equation (29) was found to be a function of the initial concentration of the AI, as changing the initial concentration alters *θ*_0_ (see Table 1).

The deliquescent droplet volume, *V*_Del_, is the minimum value that the volume of water in the droplet, $V_{{\text{H}}_{2}O}^{\text{drop}}\left(t\right)$, can take at a given point in time, concentration of AI in the droplet, *c*_AI_(0, *t*), POD and relative humidity. We find *V*_Del_ with:

(30) $$\begin{array}{lll} V_{\text{Del}}\left(t\right) & =\frac{m_{\infty }\left(\Phi \right)\;M_{\text{w,AI}}\;c_{\text{AI}}\left(0,t\right)\;V_{{\text{H}}_{2}O}^{\text{drop}}\left(t\right)}{\rho_{{\text{H}}_{2}O}}, & t>0, \end{array}$$

where the deliquescent droplet volume, *V*_Del_, is described by the mass ratio of water absorbed per mass of CaCl 2 applied, *m*_∞_, the adjusted relative humidity, Φ, the molecular weight of AI, *M*_w, AI_, the concentration of AI at the cuticle surface in the drop, *c*_AI_(0, *t*), the volume of water in the drop, $V_{{\text{H}}_{2}O}^{\text{drop}}\left(t\right)$ and the density of water, ρ_H_2_*O*_.

The maximum concentration of an ionic solution at a certain relative humidity and POD, *c*_POD_, is modeled. Equation (1) for *c*_mass%_ can be used to find *c*_POD_ as follows:

(31) $$\begin{array}{ll} c_{\text{POD}} & =\frac{c_{\text{mass}\%}\left(\Phi \right)\;\rho_{\text{AI}}}{100\%\;M_{\text{w,AI}}}, \end{array}$$

where *c*_POD_ is the maximum concentration that the AI can reach before crystallization occurs as a function of the shifted humidity, Φ, *c*_mass%_ is the final weight percentage of the AI, ρ_AI_ is the density of the AI and *M*_w, AI_ is the molecular weight of the AI.

The concentration of water in the droplet can be less than in the cuticle. The concentration at the bath boundary condition for water is constant, as shown in Equation (19). This constant is the maximum value that the concentration of water can reach. If the concentration of water is less than pure water in the cuticle or cuticle surface, water will diffuse from regions of high to low concentration. At the cuticle surface boundary condition within the drop in Equation (18), the water concentration is slightly less than pure water due to the presence of AI. Diffusion of water can occur from the bath to the drop minutely, causing the droplet volume to increase. To account for this situation and conserve mass, an additional flux term for water is added, as seen in the third line of Equation (28). This term has the same constants as the flux term in the droplet boundary condition for AI in Equation (20), except the term is also converted to a volume in time by multiplying by the molecular weight of water, *M*_w,_H__2_*O*_, and dividing by the density of water, ρ_H_2_*O*_.

If the relative humidity is below the POD of an AI with surfactant, the solution evaporates as follows:

(32) $$\begin{array}{l} \frac{dV_{{\text{H}}_{2}O}^{\text{drop}}}{dt}=-\pi \;\Lambda \;f\left(\theta_{\text{rec}}\right){\left(\frac{3\;g\left(\theta_{\text{rec}}\right)\;V_{{\text{H}}_{2}O}^{\text{drop}}\left(t\right)}{\pi }\right)}^{\frac{1}{3}}\\ +\frac{\eta_{\text{pore}}\;A_{\Pi }\;M_{\text{w},{\text{H}}_{2}O}\;A_{\text{drop}}\left(t\right)}{\rho_{{\text{H}}_{2}O}}\left[D_{{\text{H}}_{2}O}\left(x,t\right)\frac{\partial }{\partial x}\left(\varepsilon \left(x,t\right)c_{{\text{H}}_{2}O}\left(x,t\right)\right)\right]{|}_{x=0},\\ H\leq \text{POD},\;t>t_{\text{rec}}, \end{array}$$

which is the same as Equation (28), except it does not include hygroscopic growth.

To summarize, there are two parameters that changed within the model when RSO 5 is added. Firstly, the ξ adjustment factor, which alters the POD. It affects the maximum concentration AI can reach, *c*_POD_ and the maximum water absorption, *m*_∞_, which impacts *V*_Del_ and evaporation rate. Secondly, the *θ*_0_, which changes the area of the drop, number of aqueous pores under the drop and evaporation rate - refer to Table 1. When the type or concentration of adjuvant is changed within the model, the following parameters change: ξ, *θ*_0_ and scaling factor of 12.64% that used to calculate *θ*_rec_ from *θ*_0_, which impacts when the evaporation modes change.

### 2.4. Penetration Calculation

The output of our model is the concentration of AI, *c*_AI_ and water, *c*_H_2_*O*_, that has traveled through the cuticle to the water bath. Experiments measure penetration, which is the cumulative mass of AI in the water bath. We have formulated the penetration of calcium (Ca) as a mass, *m*(*t*) (in μg), at the water bath as follows:

(33) $$\begin{array}{l} m\left(t\right)=-\;10^{6}\;M_{\text{w,AI}}\;\eta_{\text{pore}}\;A_{\Pi }\;n_{\text{drops}}\\ \times \overset{t_{\text{final}}}{\underset{0}{\int }}\;A_{\text{drop}}\left(t\right)\;\left(D_{\text{AI}}\left(x,t\right)\;\frac{\partial \left(\varepsilon \left(x,t\right)c_{\text{AI}}\left(x,t\right)\right)}{\partial x}\right){|}_{x=b}\;dt, \end{array}$$

where the constant 10^6^ converts from g to μg and the flux at the bath boundary is integrated over time, where *t*_final_ is the experiment duration. We convert the mass penetration, *m*(*t*), to a percentage as follows:

(34) $$\begin{array}{ll} \%\text{Ca penetration}\left(t\right) & =\frac{m\left(t\right)}{10^{9}\;c_{AI,0}^{drop}\;n_{\text{drops}}\;V_{0}}\times 100\%, \end{array}$$

where *m*(*t*) is the mass penetration in μg from Equation (42), 10^9^ converts from μg to g and m^3^ to L, $c_{AI,0}^{drop}$ is the initial applied concentration of AI in g/L, *n*_drops_ is the number of individual drops applied to the cuticle surface (5) and *V*_0_ is the initial droplet volume in m^3^.

### 2.5. Dimensionless Model

The plant cuticle model as described in Equations (3 - 33) can be scaled and simplified using dimensionless parameters. This allows a sensitivity analysis to be performed, which is discussed in Section 3.1. For completeness, the full dimensionless model is shown in the Supplementary Materials.

### 2.6. Numerical Solution Procedure

The dimensionless model, as described in the Supplementary Materials, is solved numerically. This is achieved with a finite volume method by discretizing the model's partial differential equations using second order central differences to approximate the spatial derivatives and averaging of the diffusivity function at the control volume faces with evenly distributed nodes (Grasselli and Pelinovsky, 2008, Chapter 6). The resulting system of ordinary differential equations is then solved using “ode15i” (Shampine, 2002) within MATLAB® (MATLAB, 2017) on a desktop computer. The parameters *F*_*S*_, *k*, χ and η_pore_ were found by using optimization heuristics of a Nelder-Mead algorithm (Lagarias et al., 1998) with “fminsearch” and a Fedorov exchange algorithm (Fedorov, 1972), first with 5 μg, then across all five initial applied concentrations, for both CaCl 2 and CaCl 2 with RSO 5.

## 3. Results

The dimensionless plant cuticle model, as described in the Supplementary Materials, is solved numerically. The fitting exercise provided a fit within the error bars of the experimental data across 10 graphs for both CaCl2, and CaCl2 with RSO 5. The parameters are described in Table 2. We compare the numerical results of the CaCl2 with RSO 5 model to the experimental data from Kraemer et al. (2009). All parameters used, *F*_*s*_, η_pore_, *k* and χ are the same across all 10 graphs, with the exception of the initial applied concentration of AI, $c_{\text{AI,0}}^{\text{drop}}$. CaCl 2 is applied at 1, 5, 10, 15, and 30 g/L and each cuticle receives five 1μL drops totaling 5, 25, 50, 75 or 150 μg of Ca per cuticle. The mean mass penetration at 48 h and mean R-squared values are shown in Table 3.

**Table 3:** Comparison of the R-squared values for validation.

**Surfactant**	**Figure**	**R-squared–%**	**Mean penetration–%**	**SD–%**
RSO 5	Data	–	56.2	8.4
RSO 5	Figure 4	90.4	53.7	5.1
–	Data	–	41.8	5.7
–	Figure 5	83.6	32.6	4
Combined	Figures 4, 5	87	–	–

*The mean mass percentage penetration at 48 h with standard deviation (SD) compared with experimental data (Kraemer et al., 2009) is also shown. All values without RSO 5 exclude the outlier (B) for 25 μg*.

**Figure 4:**
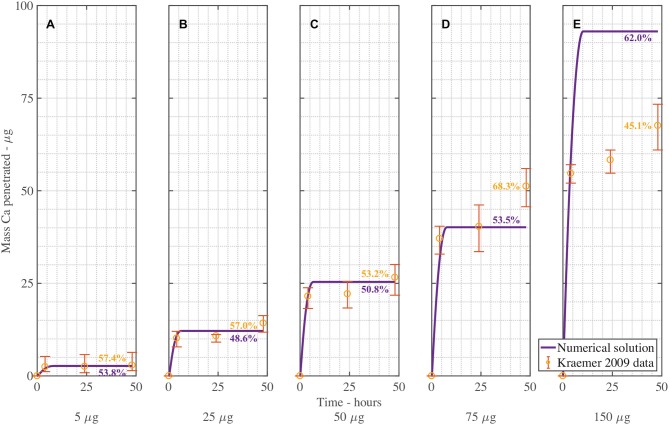
Numerical solution of the model compared to experimental data (Kraemer et al., 2009) of CaCl 2 with RSO 5, using an applied mass of Ca of 5 μg **(A)**, 25 μg **(B)**, 50 μg **(C)**, 75 μg **(D)**, 150 μg **(E)** over 48 h and parameters outlined in Table 2. The numerical solution can be seen as the continuous purple line and the experimental data as orange circles with error bars. The final percent Ca penetration is shown on each subfigure at 48 h.

Figure 4 shows the validation of the CaCl2 with RSO 5. In Figure 4, the numerical solution fits the data very well, as shown in Table 3, with the exception of Figure 4E. When comparing the mean mass penetration at 48 h, the numerical results compare very well. As seen in Figure 4, the penetration increases significantly over the first 10 h, then levels out and approaches a maximum value.

The mean percentage penetration at 48 h is 53.7% across all numerical solution results in Figure 4. As penetration has leveled out at less than 100%, it cannot increase further, unless an additional mechanism is identified. Penetration levels out due to the concentration of AI in the droplet reaching zero. Penetration levels out at less than 100% due to ion binding. In this case, ion binding causes 46% of the ions to be trapped, so is significant.

The fit in Figure 4 is reasonable, with the exception of Figure 4E for 150 μg. Numerical tests reveal that this is largely due to the value of the ion binding term. When the parameters *F*_*s*_ = 1.138, *k* = 3.74e−15, η_pore_ = 1.99e15 and χ = 0.0023 are used, the fit improves. Across all 5 graphs with RSO 5, an R-squared value of 96.2% is obtained, including the error bars. However, implementing this change over all 10 graphs including CaCl2 alone does not improve the fit, so this parameter change is not retained.

**Figure 5:**
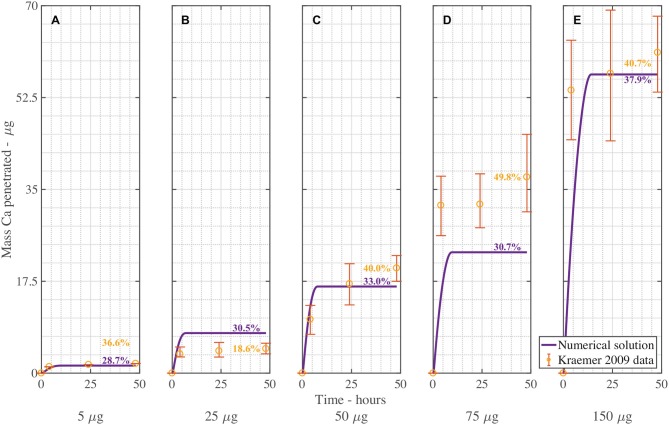
Numerical solution of the model compared to experimental data (Kraemer et al., 2009) of CaCl 2 (without RSO 5), using an applied mass of Ca of 5 μg **(A)**, 25 μg **(B)**, 50 μg **(C)**, 75 μg **(D)**, 150 μg **(E)** over 48 h with parameters outlined in Table 2. The numerical solution can be seen as the continuous purple line and the experimental data as orange circles with error bars. The final percent Ca penetration is shown on each subfigure at 48 h.

Figure 5 is the validation of the numerical results of the model compared with data for CaCl 2 (without RSO 5). Figure 5 produced a fit as seen in Table 3, excluding Figure 5B. Figure 5B is excluded as the total mean penetration for all the subfigures in Figure 5 excluding Figure 5B is 42% with a standard deviation of 6%. However, in the experimental data of Figure 5B, the penetration is only 19%, so may be an outlier and no explanation is provided (Kraemer et al., 2009).

Overall, the validation of the plant cuticle model with CaCl 2, and CaCl 2 with RSO 5 has produced reasonably good results, where one set of fitted parameters can produce fits over both data sets.

We note the experimental time is 48 h in Figures 4, 5. The numerical results for validation in these figures has been extended to 48 h for comparison with experimental data, while selected subsequent figures do not have the extended solution.

Figure S2 shows the results of AI diffusion and water diffusion with adsorption, through the plant cuticle in time. An initial concentration of AI of 1 g/L or 5 μg was used in both figures and parameters described in Table 2. The droplet of ionic solution is located at *x* = 0 and the water bath is located at *x* = 1.87 × 10^−5^ m. In Figure S2A we see the concentration profile of AI. The initial applied concentration of AI at the droplet, at *x* = 0, is 9 mol/m^3^, which quickly increases to 200 mol/m^3^ at 4.3 h, then decreases back to zero at 9.4 h. The initial rapid increase at the cuticle surface is due to the droplet solution becoming more concentrated due to rapid droplet evaporation in the first hour. Then the concentration of AI in the droplet decreases again as AI is transported into the cuticle via diffusion and ions are lost to ion binding. Within the cuticle aqueous pores, the AI is transported from regions of high concentration at the drop, to regions of low concentration at the bath, via diffusion.

Figure S2B shows the concentration profile of water. At the cuticle surface, the concentration of water in the droplet is inversely proportional to AI, as shown in Equation (18). The concentration of water at the droplet decreases further as the concentration of AI in the droplet increases due to evaporation. Then the water concentration increases again as AI concentration decreases and water diffuses in the direction from the bath to the water droplet. At late times equilibrium is reached and the concentration of water is that of pure water everywhere in the cuticle.

**Figure 6:**
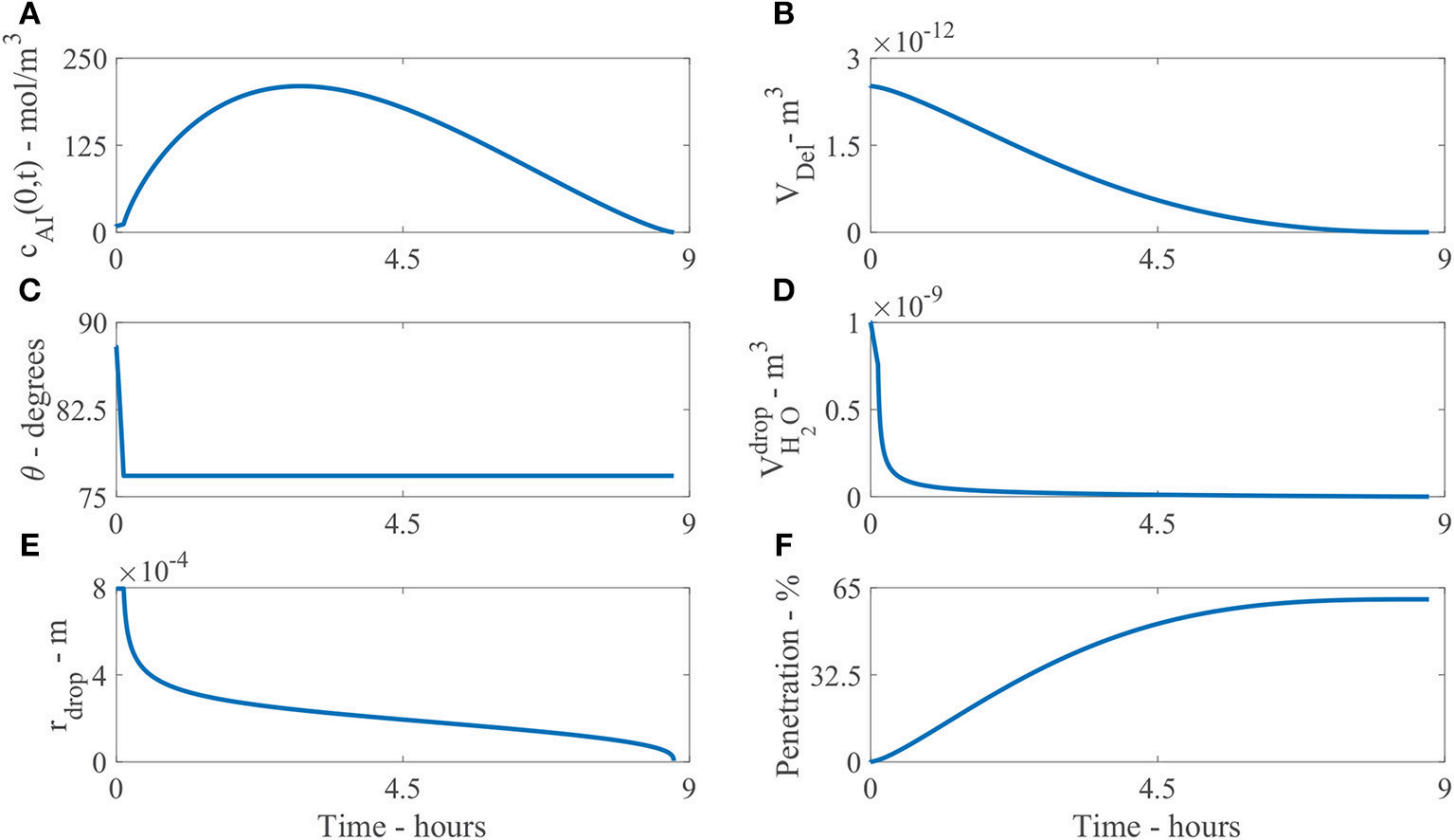
Plant cuticle model results for a single applied initial concentration of 1 g/L or 5 μg and parameters shown in Table 2. The results are all shown at the boundary where the droplet sits on the surface of the cuticle, except **(F)**. Here **(A)** shows the concentration of AI in the drop over time, *c*_AI_(0, *t*), **(B)** the deliquescence droplet volume, *V*_Del_, **(C)** the droplet contact angle, *θ*, **(D)** the volume of water in the drop, $V_{{\text{H}}_{2}\text{O}}^{\text{drop}}$, **(E)** the droplet radius, *r*_drop_ and **(F)** the percentage mass penetration of AI.

Figure 6 shows the numerical model results of a single initial applied concentration of AI of 9 mol/m^3^ and parameters shown in Table 2. The results are all shown at the droplet boundary condition, at *x* = 0, on the cuticle surface. Figure 6A shows the concentration of AI in the drop over time, *c*_AI_(0, *t*). Initially there is 9 mol/m^3^, then as time progresses, the concentration of AI in the droplet quickly increases due to rapid initial evaporation. The concentration then decreases as AI is transported through the cuticle via diffusion or is lost to ion binding over several hours.

Figure 6B shows the deliquescence droplet volume, *V*_Del_ over time. *V*_Del_ initially starts out at a large value as the initial droplet volume is large, then decreases as the water in the droplet, $V_{{\text{H}}_{2}O}^{\text{drop}}$, decreases.

Figure 6C shows the droplet contact angle, *θ*, over time. The evaporation starts in CCR mode, then continues in CCA mode. The contact angle changes from *θ*_0_ to *θ*_rec_. In Figure 6C we can see the change is not significant, as *θ*_rec_ is 12.64% less than *θ*_0_. The initial contact angle *θ*_0_ is based on experimental data for the initial contact area, explained around Table 1. However, it is not clear at exactly what time the area was measured (initially or a few seconds or minutes later after droplet deposition). As discussed around Figure 1, the evaporation starts a few minutes after droplet deposition, when the spreading phase has ceased. Further experimental work could be carried out to explore the evaporation stages for CaCl 2 with RSO 5 on tomato fruit cuticles.

Figure 6D shows the volume of water in the droplet, $V_{{\text{H}}_{2}O}^{\text{drop}}$, over time. The evaporation is initially rapid over the first hour, then after 1 h, the evaporation rate has reduced due to water absorption and POD effects governing the equation. The evaporation rate continues to reduce smoothly and the droplet completely evaporates at 9.4 h. Without the input of hygroscopic water absorption in the droplet, numerical tests show the drop would completely evaporate at 40 min and penetration would cease as there is no water in the droplet. Therefore, the water absorption effects have promoted a significant extension to the penetration timescale from 40 min to 9.4 h, allowing further penetration through the cuticle.

Figure 6E shows the droplet radius, *r*_drop_, over time. After the model has been solved numerically, the droplet volume, $V_{{\text{H}}_{2}O}^{\text{drop}}$, and contact angle, *θ*, are known. The droplet radius can then be calculated with Equation (24) to plot Figure 6E. As the evaporation mode starts at CCR mode, the radius is initially constant, then evaporation continues as CCA mode until the droplet completely evaporates and the radius decreases until penetration ceases. This change in the droplet radius is dictated by the droplet volume from Equation (28). Figure 6F shows the percentage mass penetration over time. Here we can see penetration is rapid over the first 5 h, then after 5 h, the penetration rate decreases and ceases at 9.4 h.

**Figure 7:**
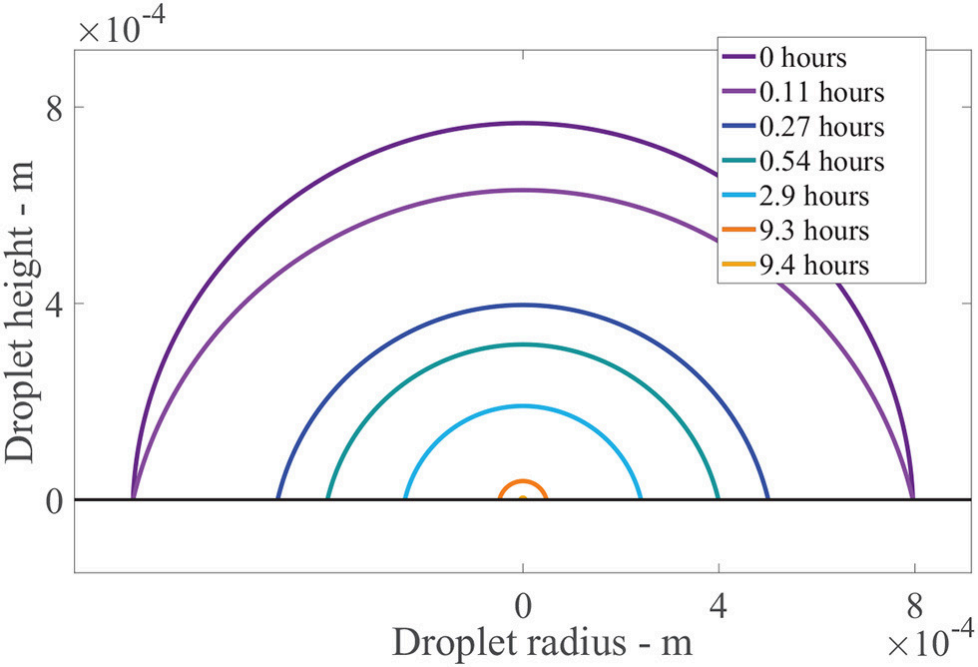
Droplet evaporation profile results for the plant cuticle model. An evaporating sessile droplet of a spherical cap shape are shown as a cross-section, changing in time, on the cuticle surface. A single applied initial concentration of 1 g/L or 5 μg is used and parameters shown in Table 2. The cuticle surface is shown as the horizontal black line. The radius of the drop and droplet height are shown in meters.

Figure 7 shows the results of the change in the droplet profile, over time. The sessile droplet with a spherical cap geometry sits on the outer cuticle surface (black line). When the model is solved numerically, the droplet radius and contact angle are known at every point in time. The droplet can then be plotted using spherical cap geometry. The radius of the whole sphere containing the spherical cap, *R*_*s*_ = *r*_drop_/sin(*θ*) (Erbil, 2012) and *θ* are converted to polar coordinates and plotted. In Figure 7, initially a drop with an initial contact angle, *θ*_0_, and radius, *r*_drop, 0_, sits. The drop then evaporates in CCR mode until *t*_rec_ is reached at 0.11 h, where we can see the angle has changed but not the radius. Then the evaporation mode changes to CCA mode where the radius changes but the contact angle stays constant. The droplet radius rapidly decrease over the first hour. Then to 9.3 h, evaporation reduces significantly due to hygroscopic effects. At around 9.4 h, the droplet has completely evaporated. This figure shows the evaporation modes and the impact the area under the drop has, which is changing in time. There is a substantial decrease in the area under the drop and consequently the number of aqueous pores under the drop in the first hour, which reduces the penetration rate.

### 3.1. Sensitivity Analysis

A sensitivity analysis was performed with the results from the dimensionless model. We have used values for CaCl2 with RSO 5 given in Table 2 with $c_{\text{AI,0}}^{\text{drop}}=1$ g/L. The one-factor-at-a-time method has been utilized to determine parameter sensitivity (Saltelli et al., 2008). A selection of parameters having the highest to lowest effect on percentage penetration are shown in Table 4. Further details can be found in Supplementary Materials Section 4 and Tredenick et al. (2017). A selection of sensitivities are discussed below.

**Table 4:** Sensitivity rankings of parameters based on the one-factor-at-a-time method, calculated in 3 ways - percentage relative sensitivity at the final time (48 h), S_%_, and the sensitivity based on the residual sum of squares, SS_res_ and root-mean-square error, RMSE over time, S_SS_res__ and S_RMSE_.

**Parameter**	**Description**	**S_***%***_**	**S_**SS_res_**_**	**S_**RMSE**_**	**Proportionality**
*F*_*s*_	Fractal scaling - tortuosity	1	1	1	Inverse
$r_{p}^{max}$	Maximum aqueous pore radius	2	2	2	Direct
*H*	Relative humidity	3	4	3	Direct
η_pore_	Aqueous pore density	4	3	4	Direct
ξ	POD RH adjustment factor for RSO 5	5	6	6	Direct
*θ*_0_	Initial droplet contact angle	6	5	5	Inverse
*k*	Ion binding	7	8	8	Inverse
*b*	Cuticle thickness	8	7	7	Inverse
χ	Droplet evaporation scaling factor	9	9	9/10	Inverse
*V*_0_	Initial droplet volume	10	10	9/10	–
$c_{\text{AI,0}}^{\text{drop}}$	Initial AI droplet concentration	11	11	11	–

*Additional details can be found in Supplementary Materials section 4*.

#### 3.1.1. Fractal Scaling Dimension and Relative Humidity - *F*_*s*_ and *H*

**Figure 8:**
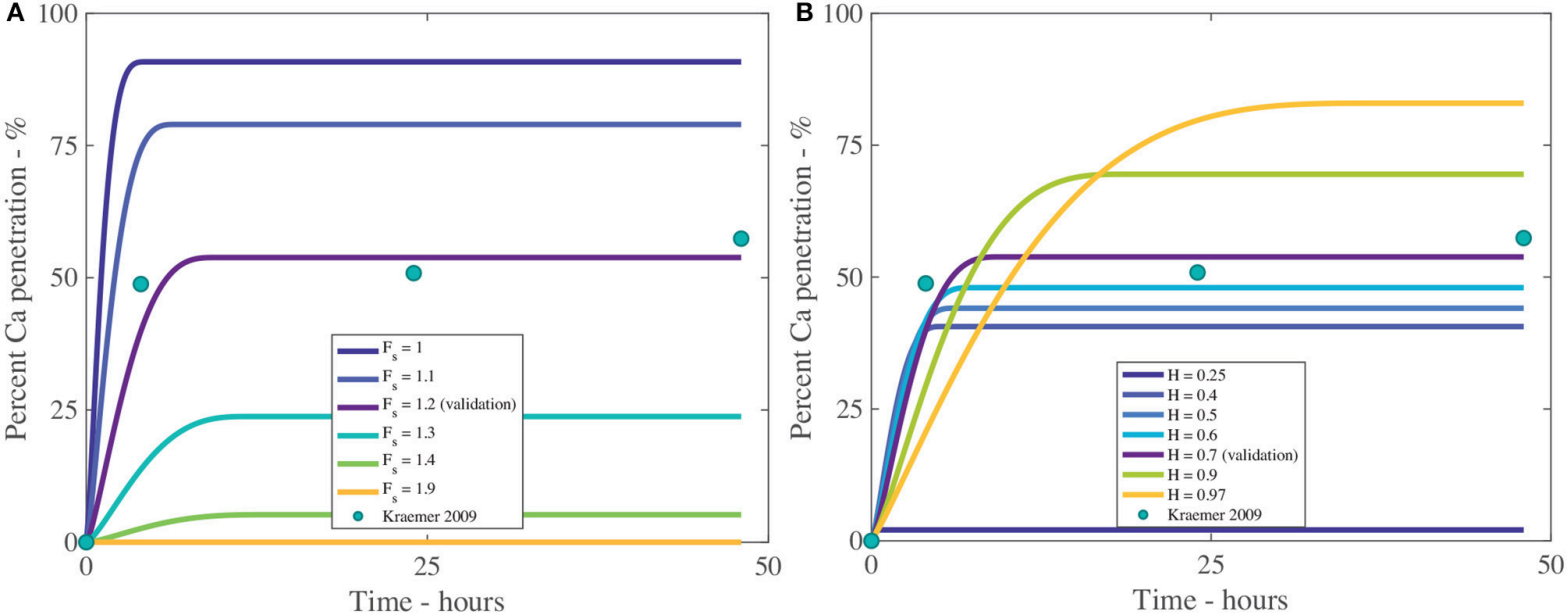
Percent calcium (Ca) penetration sensitivity to *F*_*s*_ **(A)** and *H* **(B)**, with parameters described in Table 2. The reference case, used for validation, can be seen in purple and the experimental data as green circles.

**Figure 9:**
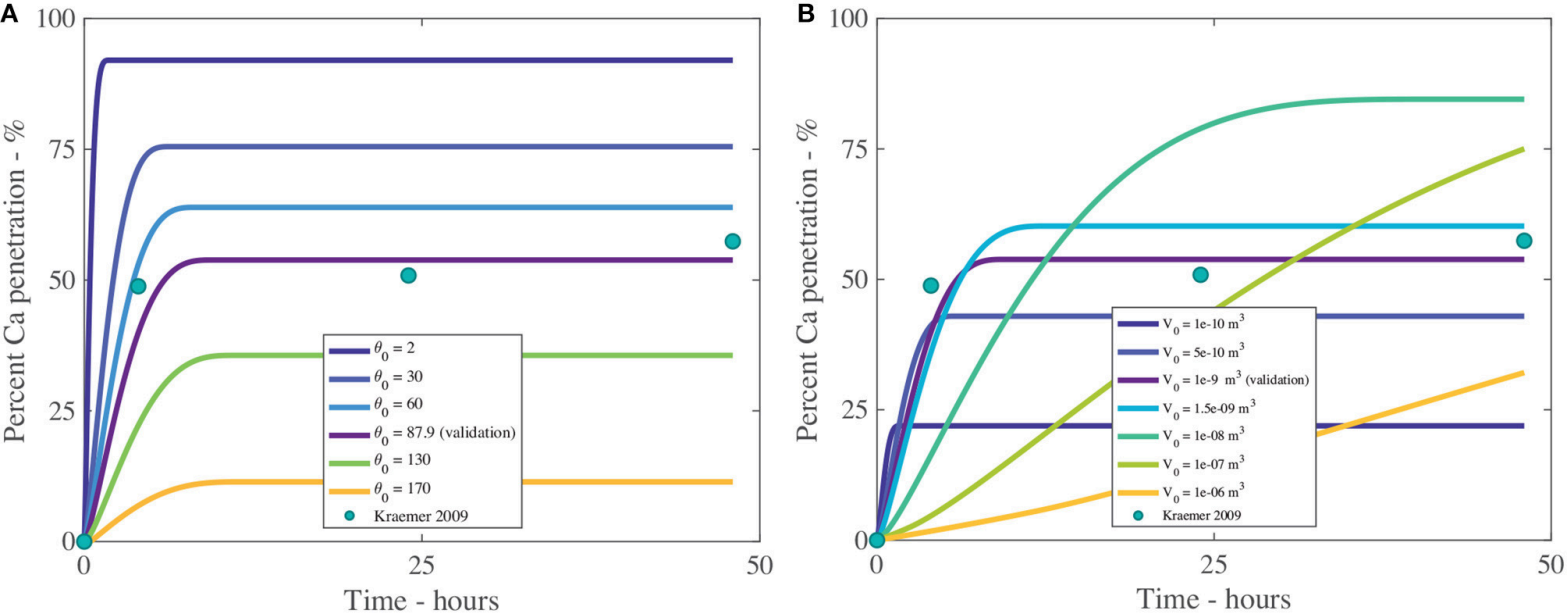
Percent calcium (Ca) penetration sensitivity to *θ*_0_ **(A)** and *V*_0_ in m^3^ **(B)**, with parameters described in Table 2. The reference case, used for validation, can be seen in purple and the experimental data as green circles.

The fractal scaling dimension for the tortuosity of the aqueous pores, *F*_*s*_, influences penetration to an extremely high level, as shown in Table 4 and Figure 8A. In Figures 8, 9, the reference case, used for validation, can be seen in purple and the experimental data as green circles. In the model, *F*_*s*_ impacts the effective diffusivity of the AI and water. As seen in Figure 8A, changing *F*_*s*_ alters the timescale of penetration. At a *F*_*s*_ of 1, penetration can rapidly reach 90%, while at *F*_*s*_ of 1.9, penetration is close to zero. In isolated cuticles, the tortuosity of the aqueous pores will vary between plant species to a significant degree due to structural changes like lamellate structures, thickness, orientation of pores and plant age (Santier and Chamel, 1992; Schreiber et al., 2006; Jeffree, 2008; Kerstiens, 2010). The large effect *F*_*s*_ has over penetration may explain the differences seen in penetration in different plant species, which is consistent with experimental work (Schreiber et al., 2006).

In Figure 8B we can see the sensitivity of our model to the relative humidity, *H*. As humidity increases, the maximum percentage penetration also increases and is highly influential over penetration, as seen in Table 4. Relative humidity changes the timescale of penetration. In the model, relative humidity affects penetration by influencing the evaporation rate, the POD, the maximum concentration of the solution and the water absorption over the AI.

In Figure 8B, at high humidities, such as 90%RH or 97%RH, the highest penetration is obtained. High relative humidity promotes high penetration as the droplet evaporates at a slower rate, hence the droplet is larger, covering a larger surface area and hence a larger number of pores under the droplet, extending the penetration time.

When relative humidity is close to saturation at 97%RH, 84% penetration is achieved. As penetration has leveled out and ceased at less than 100% penetration, this indicates some ions are lost to ion binding (16%), but less than at 70%RH (45% loss to ion binding). This is due to a linear relationship with the concentration of AI in the droplet in Equation (20). At high humidities, the droplet stays dilute for longer as the evaporation rate is reduced, so less ions are lost to ion binding. This indicates there is a balance that needs to be found between humidity and concentration of AI in the droplet.

In Figure 8B, the lowest penetration is obtained at 25%RH, reaching 2% at 16 min. The POD_AI_ of CaCl_2_ is 32%RH, which is above the humidity of 25%RH, which means AI does not absorb water and the droplet evaporates according to Equation (32). The penetration ceases when there is no water left on the cuticle surface or the concentration of AI is zero. The water evaporates very quickly as the evaporation rate has increased due to the low relative humidity and there is no water absorption. This has caused penetration to end at 16 min compared to 35 h with 97%RH and much lower penetration. Relative humidity has a significant impact and shows the importance of the POD.

In isolated cuticles, penetration has been found to increase significantly with relative humidity (Schönherr, 2000, 2002). In Figure 8B this significant increase is also seen, where penetration increases exponentially as relative humidity increases. The cause of this increase is due to the exponential increase in hygroscopic water absorption over the CaCl_2_, as seen in Figure 2B. At high relative humidities, the water absorption is very high, which produces high penetration in Figure 8B. Several studies have investigated ionic AI penetration with adjuvants under various relative humidities through isolated cuticles but are rarely with RSO 5. These studies are worth considering nonetheless. Several studies have shown relative humidity has a significant impact on penetration with and without adjuvants and is directly proportional (Middleton and Sanderson, 1965; Schönherr, 2000, 2002, 2006). These results indicate our model has the potential to predict changes in relative humidity, beyond the Kraemer et al. (2009) data and are in line with the well-established literature.

#### 3.1.2. Initial Droplet Contact Angle and Initial Droplet Volume - θ_0_ and *V*_0_

The effect that the initial contact angle, θ_0_, has on penetration can be seen in Figure 9A and Table 4. The sensitivity analysis reveals that θ_0_ is highly influential over penetration, having an inverse relationship to penetration. Within the model, θ_0_ has the most influence over penetration by changing the initial contact area under the droplet, which changes the number of pores available to diffusion. Decreasing the contact angle is one way surfactants influence the droplet. In Figure 9A, penetration still increases with decreasing initial contact angle, despite this decreased penetration time and increased evaporation rate, due to the balance that is obtained by increasing the droplet contact area, the number of pores under the drop and the AI in the droplet becoming more concentrated earlier, promoting penetration. This increased evaporation rate of low flat drops produced using surfactants is also seen in the literature, where a thinner liquid layer is created. This thin layer promotes heat transfer from the solid to the liquid-vapor interface. The contact area of the droplet also increases the heat transfer area. These two effects promote surface cooling, which promotes droplet evaporation (Chandra et al., 1996).

The initial contact angle changes with type and concentration of adjuvant (Haefs et al., 2002; Xu et al., 2011) and plant species. Some plant species are easy to wet while others are difficult to wet, based on surface structures such as trichomes, stomata and cuticle folds. Here the results of the sensitivity analysis align with the well-established literature and allow for the inclusion of many different plant species and adjuvant into the model.

The initial volume of the droplet, *V*_0_, as shown in Figure 9B and Table 4, has a large impact over penetration. We note only *V*_0_ has changed, not the initial contact angle or initial concentration of AI. The initial droplet radius and area, which are calculated, do change. In Figure 9B, we can see that increasing the initial volume increases percentage penetration and alters the timescale of penetration. However, at large volumes, the penetration is not as high. In Figure 9B, a *V*_0_ of 1 × 10^−8^ m^3^ reaches 90% penetration quickly and seems to be the optimal value here. In Figure 9B, we can see that a small initial volume of 1 × 10^−10^ m^3^ reaches 20% penetration and ceases very quickly. The droplet is so small that evaporation is very rapid. This causes the droplet to become concentrated very quickly, which increases the concentration gradient through the cuticle, promoting rapid penetration and ion binding.

An initial volume of 1 × 10^−6^ m^3^ is the largest value tested, however it has not yet reached its maximum penetration. If the timescale is increased to 150 h, close to 100% penetration is obtained using the two largest initial volumes. It would appear that there is a balance that needs to be achieved with the initial droplet volume. Evaporation will be much slower for a larger droplet and it will become highly concentrated at a much later time. A balance needs to be found where the droplet is small enough to cause some evaporation and increased droplet concentration, but not too small otherwise the evaporation is too rapid and penetration quickly ends.

When the initial volumes are applied to sprays, they correspond to spherical droplets in air of diameters of 600, 980, 1,400, 2,600, 6,000, and 12,400 μm. Sprays are typically 60–600 μm and large rain is up to 4,000 μm (ASABE, 2009), so the two largest volumes in Figure 9B are unlikely to exist in nature.

In isolated cuticles, the low penetration with a large droplet size and the balance that needs to be found could possibly explain why certain ionic AIs such as glyphosate can require a concentrated droplet to promote penetration. Santier and Chamel (1992) studied glyphosate (without adjuvants) through isolated tomato fruit cuticles and found that at 100%RH, penetration does not occur until after 20 h when the droplet has mostly evaporated, then penetration can reach 90%.

#### 3.1.3. Point of Deliquescence

We investigate the sensitivity of the model to the point of deliquescence, POD. We investigate the POD of other possible ionic AIs. We note that the water absorption data for other ionic AIs, *c*_mass%_ and *m*_∞_, would vary. When the ionic AI or surfactant changes, producing a new POD, new experimental data can be found for *c*_mass%_ and *m*_∞_, which can then be fitted with a function.

To find the POD of an ionic AI solution with a surfactant, we consider how much change this has applied to the POD of the ionic AI solution. Here we consider large changes to the POD, so large values of ξ, the POD adjustment factor, as seen in Table S1. To then find the resulting values for *c*_mass%_ and *m*_∞_, we find the value of the independent variable, Φ, which is based on the relative humidity, *H*, the change in POD, ξ, and the ionic AI solution POD without surfactant, POD_AI_ (32%RH for CaCl_2_ Kolthoff et al., 1969), as follows:

(35) $$\Phi =\;\{\begin{array}{ll} H+\xi & :H+\xi >{\text{POD}}_{\text{AI}},\\ 97\%\; & :H+\xi >97\%. \end{array}$$

In Equation (35), if *H* + ξ is larger than POD_AI_, water absorption can occur. If *H* + ξ is larger than 97%RH, Φ is set to 97%RH. If *H* + ξ is less than or equal to POD_AI_, *c*_mass%_ and *m*_∞_ are not included in evaporation and penetration ceases when there is no water in the droplet or the concentration of AI has reached zero.

**Figure 10:**
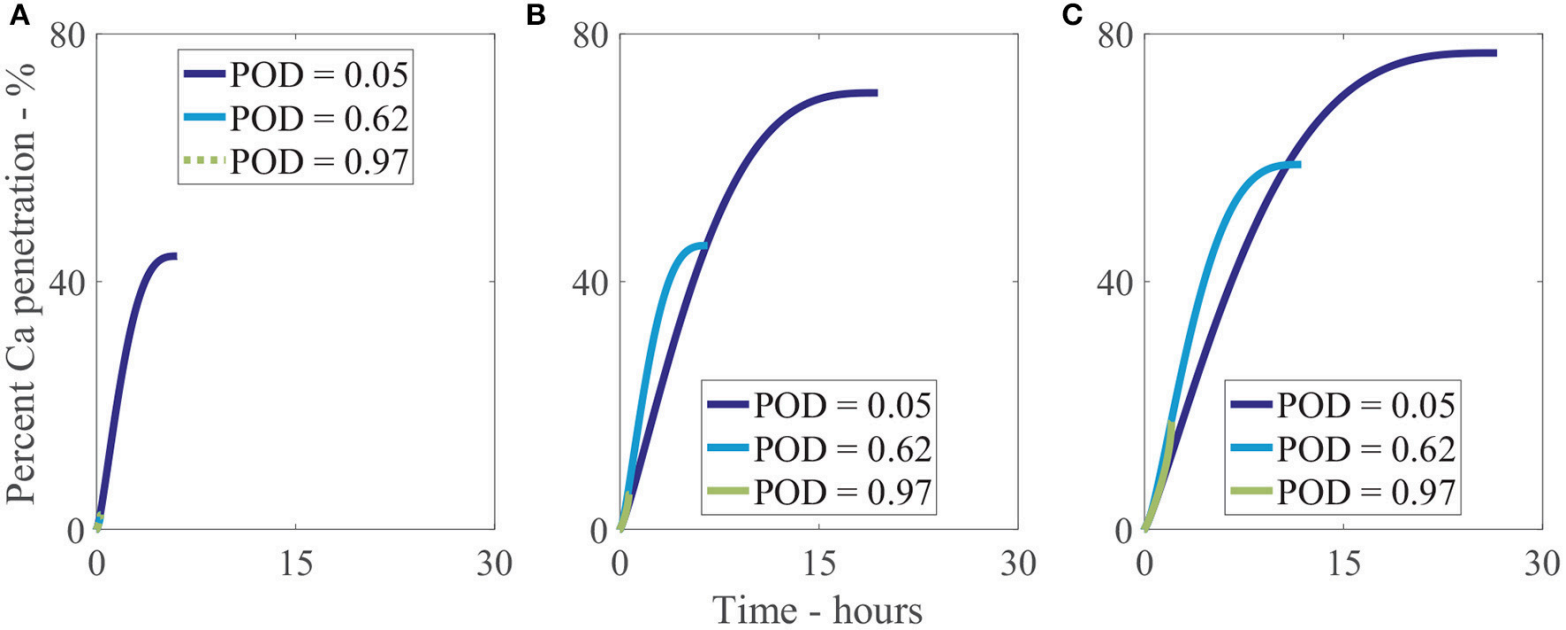
Percent calcium (Ca) penetration sensitivity to the POD of a solution. Relative humidity is 33%RH **(A)**, 70%RH **(B)**, and 90%RH **(C)**. Parameters are described in Table 2.

We have investigated 3 different relative humidities with 3 different POD values for each, as shown Figure 10. In Figure 10A, at a low relative humidity of 33%RH, only a very low POD of 5%RH can allow water absorption and hence increased penetration to occur, reaching 40% penetration at 5 h. The penetration at a POD of 5%RH is 19-fold that of the penetration at a POD of 62%RH and 97%RH at the final time or a relative change of 44%. In Figure 10B, at 70%RH, higher water absorption is attainable, increasing final penetration time and penetration. The highest penetration is 13-fold that of the lowest or a relative change of 68%. The effect is similar in Figure 10C, at 90%RH, where the highest penetration is 4-fold that of the lowest or a relative change of 63%. Figures 10A–C shows the significant effect POD with relative humidity has on penetration and is a significant limiting factor, with a 19-fold difference in some cases. In isolated cuticles, we surmise this POD limiting factor is one of the reasons very low penetration is seen in some cases without adjuvants (Santier and Chamel, 1992), significantly limiting penetration.

#### 3.1.4. Other Parameters - η_pore_, *b*, $c_{\text{AI,0}}^{\text{drop}}$, ξ

The aqueous pore density, η_pore_, controls the porosity of the cuticle. As seen in Table 4, the pore density is very highly influential over penetration, it changes the timescale of penetration and is directly proportional. The parameter η_pore_ would change when plant species is changed or perhaps growing conditions (Karbulková et al., 2008) or leaf age (Viougeas et al., 1995).

The thickness of the cuticle, *b*, is highly influential over penetration, changes the timescale of penetration and is inversely proportional. The thickness of the cuticle alone is changed and the remaining characteristics of the cuticle remain the same, so the tortuosity and pore radius remain the same, therefore the diffusion path length is increased (Riederer and Schreiber, 1995), which decreases penetration. Changing cuticle thickness is one way to incorporate plant species variation. Ionic AIs take longer to diffuse through the pores of thicker cuticles, which is supported by the literature (Santier and Chamel, 1992). The inverse proportionality and sensitivity of cuticle thickness is in line with the well-established literature.

The initial concentration of AI, $c_{\text{AI,0}}^{\text{drop}}$, has moderate impact over the penetration as seen in Table 4. The proportionality of $c_{\text{AI,0}}^{\text{drop}}$ over penetration is nonlinear, as it influences many aspects within the model. Changing $c_{\text{AI,0}}^{\text{drop}}$ leads to choosing an alternative θ_0_, as shown in Table 1. In isolated cuticles, the penetration of CaCl_2_ with and without adjuvants has been shown to have only a slight dependence on the initial concentration of CaCl_2_ (Schönherr, 2000; Kraemer et al., 2009). Penetration in isolated astomatous *Pyrus* cuticles, at 90%RH, with an initial concentration of CaCl_2_ of 1 g/L, 2 g/L, 4 g/L and 6 g/L (with 0.2 g/L Glucopon 215 CSUP as a wetting agent) produced around 95% penetration at 100 h (Schönherr, 2000), therefore having no sensitivity to the initial CaCl_2_ concentration. The results for the initial concentration of AI align with the well-established literature.

The POD shifting parameter, ξ, describes how the POD of a solution and the relative humidity have changed with the addition of an adjuvant. We investigate this to show the model can accommodate changes in POD of ionic solutions. As shown in Table 4, ξ has a high effect over penetration and is directly proportional to penetration. When the POD is below the relative humidity, water absorption can occur over AI in the droplet due to hygroscopic effects. When the POD is above the relative humidity, penetration may be limited as water absorption is not included in the model.

To summarize, the parameters that are highly influential over penetration are related to variations in plant species. These parameters include θ_0_, $r_{\text{p}}^{\text{max}}$, η_pore_, *b*, *F*_s_ and *k*. This parameter combination could perhaps explain the significant differences in penetration that is seen in experimental work (Schreiber et al., 2006) in isolated cuticles from different plant species.

Parameters that impact the evaporation rate of *H*, θ_0_, *V*_0_, χ, POD and ξ impact penetration to a significant degree. However, this collective effect is less than that of the plant species parameters.

The individual parameters that have the most effect over penetration within the sensitivity analysis are the tortuosity, *F*_*s*_ and the maximum pore radius, $r_{\text{p}}^{\text{max}}$. This indicates cuticle structure plays the most influential role over penetration. Relative humidity, *H*, and pore density, η_pore_, also significantly impact penetration. The ion binding, *k*, relative humidity, *H*, POD adjustment factor, ξ, and POD were found to be limiters to penetration. There is an optimal value that needs to be found to obtain adequate penetration.

When changing the model between fitting CaCl_2_ with and without RSO 5, two parameters changed, ξ, which alters the POD of the solution and the water absorption of the ionic droplet solution and θ_0_. These parameters influence many interconnected aspects of the model. A 5% change of ξ has a minimal effect on penetration. Numerical analysis shows that the most significant impact of changing θ_0_, is that it varies the initial droplet contact area and the number of pores under the droplet. When including RSO 5, the initial droplet area is much larger. Then as time progresses, the area under the droplet remains much larger than without RSO 5, as the volume changes at a similar rate. The rate of evaporation is only slightly different with and without RSO 5. The area of the drop influences the rate that the AI travels into the cuticle in Equation (20) and the AI penetration in Equation (33). Therefore RSO 5 increases penetration in this case due to the change in θ_0_ and the number of pores under the drop.

The sensitivity analysis has provided some insight into why surfactants in general increase penetration. We can see from the sensitivity results for ξ and the POD, that if an AI has very little penetration without surfactant, when a surfactant is added it significantly alters the POD of the solution and penetration can increase significantly. Changing the POD of a solution will allow more water to be absorbed at lower relative humidities and delay evaporation, which increases the droplet spread area, the number of pores under the droplet and extends the penetration timescale, which increases penetration. Changing θ_0_ also increases penetration as shown in Figure 9. We surmise the effect that surfactants have of changing a solutions POD, which significantly increasing penetration, could partially explain the large increase in penetration found in some isolated cuticle experiments (Coret and Chamel, 1993; Schönherr, 2000).

## 4. Discussion

We have numerically solved our plant cuticle diffusion model and produced results, further discussed here. The model has 4 fitted parameters, *F*_*S*_, *k*, χ, and η_pore_, as described in Table 2. We will discuss the implications of the value of these parameters.

From the fitting exercise, a low value for the fractal scaling dimension (for tortuosity), *F*_*s*_, was obtained. This indicates the tortuosity of the aqueous pores in tomato fruit cuticles is in the lower range of tortuosity (tending to straight). Penetration is quite rapid, so this is logical. If a higher value for *F*_*s*_ was used, for example, 1.7, this would indicate very tortuous pores and penetration would be slow. If a certain plant species has a low diffusivity, very tortuous pores, a highly lamellate cuticle structure or a slow penetration rate, a high value for *F*_*s*_ can be chosen. Fitting *F*_*s*_ is reasonable in this model, as it facilitates the diffusion path length calculation, which currently cannot be found by a physical measurement (Riederer and Schreiber, 1995).

The aqueous pore density, η_pore_, as described in Table 2, was high. It is close to densities found elsewhere of 2 × 10^15^ m^−2^ in *Citrus aurantium* cuticles (Schreiber and Schönherr, 2009, p. 85). A high value for pore density indicates penetration would be rapid, as the experimental data indicates.

The fitted parameter for the logistic decay evaporation term, χ, as described in Table 2, multiplies the evaporation in Equation (28) for CCA mode. Dash and Garimella (2013) found there was a need to include an extra constant in CCA mode with the Popov (2005) model, which they attribute to evaporative cooling at the droplet interface and a lower effective thermal conductivity of the substrate due to air gaps present on the surface.

Ion binding of AI onto the cuticle surface has been identified here as a key mechanism. Ionic penetration through plant cuticles can level out at less than 100% (Santier and Chamel, 1992; Schönherr, 2000, 2006; Kraemer et al., 2009). However, some of these studies have been plotted on a log plot, making analysis difficult. If penetration levels out and the rate of change of penetration is zero, penetration has ceased. Here we have identified that penetration levels out at less than 100% due to ion binding. One paper within the literature has shown ion binding exists (Yamada et al., 1964), another found differences between the adaxial and abaxial leaf surfaces (Horgan, 2017) and a third found penetration was different when the inner or outer cuticle surface was utilized (Santier and Chamel, 1992). However, the Yamada et al. (1964) paper is not well represented within the current literature. There is not yet a body of conclusive evidence to support it. The experimental techniques could perhaps be improved and expanded upon to include various plant species, ionic AIs, relative humidities and adjuvants. There could be additional mechanisms that interact with the ions, the ions could remobilize once bound, the ions could bind inside the cuticle aqueous pores and the mechanism could change with relative humidity or the addition of adjuvants.

**Figure 11:**
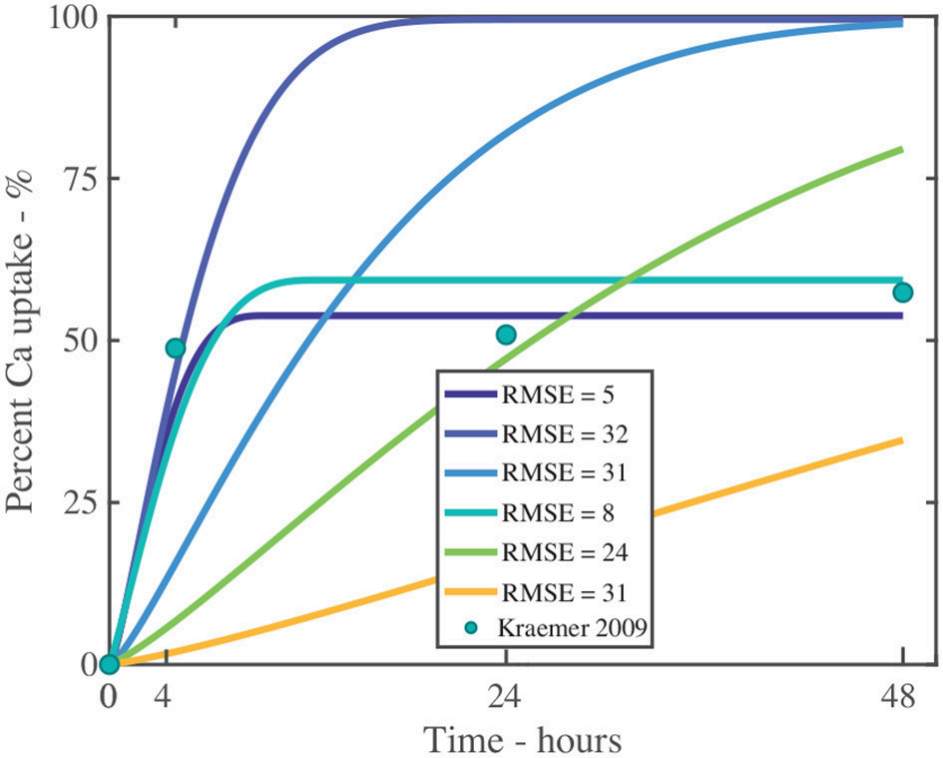
Fitting experiment both with and without ion binding for a single applied initial concentration of 1 g/L or 5 μg and parameters described in Table 2. The experimental data is shown as green circles. The additional fitting parameters are described in Table S3. The root-mean-square error, RMSE, indicates the fit, where values closer to zero indicate improved fits.

We will explore the need to incorporate ion binding into the model numerically, investigating penetration of CaCl_2_ with RSO 5 at $c_{\text{AI,0}}^{\text{drop}}\;=\;1$ g/L, as shown in Figure 11 and fitting parameters described in Table S3. We have fitted the data with 2 parameter sets. We have then removed the ion binding mechanism from the model by setting *k* to be zero. Without ion binding, as shown in Figure 11, it is not possible to fit the data and the root-mean-square errors, RMSE, are large (values closer to zero indicate better fits), as shown in Table S3. Therefore, ion binding is necessary to include within a penetration model.

Incorporating evaporation with the hygroscopic nature of ionic AIs into a mechanistic model, producing Equation (28), is a novel approach first published in this paper. This model could be useful for other circumstances such as sea salt mixed with spray formulations deposited onto foliage (Chen and Lee, 2001; Fernández et al., 2017; Lovelock et al., 2017). The evaporation model can be thought to apply to any ionic hygroscopic solution on many types of substrates, not just plant cuticles.

The plant cuticle diffusion model can theoretically apply to the penetration of most ionic hydrophilic AIs, both with and without adjuvants. It can apply to isolated astomatous plant leaf or fruit species cuticle, where the aqueous pores are sufficiently large to allow AI to be transported through the cuticle via Fickian diffusion. It can be utilized to model changes in relative humidity, AI type and concentration, plant species variation, adjuvant type and POD of AI with adjuvant. Species where transport is extremely slow is theorized to be a mechanism other than Fickian diffusion. This requires further investigation before applying the model. The model cannot be applied to lipophilic compounds, ionic hydrophilic compounds such as Fe chelates (Schönherr et al., 2005; Schlegel et al., 2006) that dehydrate aqueous pores, uncharged hydrophilic compounds or whole leaf penetration. Simple adaptations could be made to this model to account for these cases, which will be the subject of future work.

Aqueous pores are known to change in size and swell with water adsorption (Luque et al., 1995; Kerstiens, 2006; Schönherr, 2006). Swelling of the aqueous pores also occurs while penetration takes place, is included within the model and is directly proportional to the concentration of water. The initial conditions for the experimental setup (Kraemer et al., 2009) are a water droplet on the cuticle surface, a water bath on the inner cuticle surface, rehydrated cuticle and aqueous pores that are fully swelled. As there is a high concentration of water initially that does not change significantly, pores do not swell to a large degree as the pore radius is directly proportional to the concentration of water. However, under other experimental setups, pore swelling may be significant. We surmise these could include an initially dry isolated cuticle or a cuticle that was initially in a low relative humidity, then was placed in a high humidity just prior to the experiment. We leave pore swelling here as it allows the model to be more adaptable, with the view to investigate such cases in future works.

## 5. Conclusion

A mechanistic model has been developed here to simulate diffusion of hydrophilic ionic active ingredients including adjuvants through plant cuticles. We have also included diffusion and adsorption of water within the cuticle. This model makes novel additions to a simple diffusion model by incorporating the important governing mechanisms of swelling of the aqueous pores, climatic conditions such as relative humidity that affect the evaporation of water in the applied droplet. We also include parameters that account for the differences in plant species, porosity and tortuosity of the aqueous pores, ion binding to the cuticle surface, a diffusivity function that changes in time through the cuticle and parameters capable of incorporating adjuvant addition with initial contact angle and point of deliquescence adjustment. Here we have developed novel additions to the Popov (2005) evaporation model, incorporating hygroscopic water absorption and point of deliquescence effects, which may be theoretically applied to any ionic hygroscopic solution on many types of substrates, not just plant cuticles. The model has been solved numerically, producing results that show reasonable agreement with the experimental data for both ionic AI and ionic AI including surfactant, by altering several parameters, but not changing the model itself. We have discussed mechanisms of ion binding, point of deliquescence and relative humidity that significantly limited penetration and were necessary to incorporate into a penetration model. Major factors influencing penetration were found with the sensitivity analysis to be plant species variations with cuticle structure including tortuosity, porosity, maximum pore radius, pore density, initial contact angle and cuticle thickness; ion binding, evaporation and hygroscopic growth parameters such as relative humidity. The sensitivity analysis indicated surfactants increase penetration by changing the initial contact angle and changing the POD of a solution, which alters the water absorption of the solution. The results of the sensitivity analysis are in keeping with the well-established literature.

## Author Contributions

ET is responsible for article writing, model creation and adaptation, computational code creation, results, analysis, article editing and revision. TF is responsible for model creation and adaptation, results, analysis, article editing and revision. WF is responsible for motivating the project, the biological and agrochemical consultation, article editing and revision. All authors agree to be accountable for all aspects of the work in ensuring that questions related to the accuracy or integrity of any part of the work are appropriately investigated and resolved.

### Conflict of Interest Statement

The authors declare that the research was conducted in the absence of any commercial or financial relationships that could be construed as a potential conflict of interest.
