# Supplementary material for: Mathematical Modeling of Diffusion of a Hydrophilic Ionic Fertilizer in Plant Cuticles: Surfactant and Hygroscopic Effects
Source: Front Plant Sci. 2018 Dec 20;9:1888. doi: 10.3389/fpls.2018.01888 (PMC6306450; doi:10.3389/fpls.2018.01888)
Supplement: Supplementary file 1 [file Data_Sheet_1.pdf]

Supplementary Materials: Mathematical Modeling of Diffusion of a Hydrophilic Ionic Fertilizer in Plant Cuticles: Surfactant and Hygroscopic Effects

1 DIMENSIONLESS MODEL

$$\left.\begin{array}{lllll}
\bar{x} = \dfrac{x}{b}, & \bar{t} = \dfrac{D_{\mathrm{AI}}^{\mathrm{bulk}} t}{b^2}, & \overline{c_1} = \dfrac{c_{\mathrm{AI}}}{c_{\mathrm{AI},0}^{\mathrm{drop}}}, & \overline{c_2} = \dfrac{c_{\mathrm{H_2O}}}{c_{\mathrm{H_2O}}^{\mathrm{pure}}}, & \overline{D}_1 = \dfrac{D_{\mathrm{AI}}}{D_{\mathrm{AI}}^{\mathrm{bulk}}}, \\
\overline{D}_2 = \dfrac{D_{\mathrm{H_2O}}}{D_{\mathrm{H_2O}}^{\mathrm{bulk}}}, & \overline{D}_{\mathrm{total}} = \dfrac{D_{\mathrm{H_2O}}^{\mathrm{bulk}}}{D_{\mathrm{AI}}^{\mathrm{bulk}}}, & \bar{r} = \dfrac{r_{\mathrm{p}}}{r_{\mathrm{H_2O}}}, & \bar{\Gamma} = \dfrac{\Gamma_{\mathrm{H_2O}}}{\Gamma_{\mathrm{S}}}, & \mu = \dfrac{2\,\Gamma_{\mathrm{S}}}{r_{\mathrm{H_2O}}\, c_{\mathrm{H_2O}}^{\mathrm{pure}}}, \\
\bar{\beta} = \beta_{\mathrm{H_2O}}\, c_{\mathrm{H_2O}}^{\mathrm{pure}}, & \Omega = \Gamma_{\mathrm{S}}\, r_{\mathrm{H_2O}}^2\, N_{\mathrm{A}}, & \overline{V} = \dfrac{V_{\mathrm{H_2O}}^{\mathrm{drop}}}{V_0}, & \bar{\gamma} = \dfrac{\eta_{\mathrm{pore}}\, A_{\mathrm{drop},0}\, A_{\Pi}\, b}{V_0}, & \omega = \dfrac{k\, b^2}{D_{\mathrm{AI}}^{\mathrm{bulk}}\, V_0}, \\
\bar{v}_1 = \bar{v}_{\mathrm{AI}}\, c_{\mathrm{AI},0}^{\mathrm{drop}}, & \bar{v}_2 = \bar{v}_{\mathrm{H_2O}}\, c_{\mathrm{H_2O}}^{\mathrm{pure}}, & \overline{A}_{\mathrm{drop}} = \dfrac{A_{\mathrm{drop}}}{A_{\mathrm{drop},0}}, & \overline{V}_{\mathrm{Del}} = \dfrac{V_{\mathrm{Del}}}{V_0}, & \bar{t}_{\mathrm{rec}} = \dfrac{t_{\mathrm{rec}}\, D_{\mathrm{AI}}^{\mathrm{bulk}}}{b^2},
\end{array}\right\} \tag{S1}$$

$$\Lambda_2 = \frac{\eta_{\mathrm{pore}}\, A_{\mathrm{drop},0}\, M_{\mathrm{w,H_2O}}\, A_{\Pi}\, b\, c_{\mathrm{H_2O}}^{\mathrm{pure}}\, \overline{D}_{\mathrm{total}}}{V_0\, \rho_{\mathrm{H_2O}}}. \tag{S2}$$

$$\frac{\partial(\varepsilon\overline{c_1})}{\partial\bar{t}} = \frac{\partial}{\partial\bar{x}}\left[\overline{D}_1 \frac{\partial(\varepsilon\overline{c_1})}{\partial\bar{x}}\right], \qquad 0 < \bar{x} < 1,\ \bar{t} > 0, \tag{S3}$$

$$\frac{\partial(\varepsilon\overline{c_2})}{\partial\bar{t}} = \overline{D}_{\mathrm{total}} \frac{\partial}{\partial\bar{x}}\left[\overline{D}_2 \frac{\partial(\varepsilon\overline{c_2})}{\partial\bar{x}}\right] - \frac{\mu}{\bar{r}}(1-\varepsilon)\frac{\partial\bar{\Gamma}}{\partial\bar{t}}, \qquad 0 < \bar{x} < 1,\ \bar{t} > 0, \tag{S4}$$

$$\text{Functions}: \quad \bar{\Gamma}(\bar{x},\bar{t}) = \left(1 + \left(\bar{\beta}\bar{c_2}\right)^{-1}\right)^{-1}, \qquad 0 < \bar{x} < 1,\ \bar{t} > 0, \tag{S5}$$

$$\bar{r}(\bar{x},\bar{t}) = 1 + \left(\sin\left((\Omega\bar{\Gamma})^{-1}\right)\right)^{-1}, \qquad 0 < \bar{x} < 1,\ \bar{t} > 0, \tag{S6}$$

$$\varepsilon(\bar{x},\bar{t}) = \pi\left[\frac{\bar{r}\ r_{\mathrm{H_2O}}}{L}\left(\sqrt{n_0}+1\right)\right]^2, \qquad 0 < \bar{x} < 1,\ \bar{t} > 0, \tag{S7}$$

$$\overline{D}_1(\bar{x},\bar{t}) = \overline{D}_2(\bar{x},\bar{t}) = \varepsilon^{\left(\frac{F_s}{2-F_s}\right)}, \qquad 0 < \bar{x} < 1,\ \bar{t} > 0, \tag{S8}$$

$$\text{ICs}: \quad \bar{c_1}(\bar{x},0) = 0, \qquad 0 < \bar{x} < 1, \tag{S9}$$

$$\bar{c_1}(0,0) = 1, \tag{S10}$$

$$\bar{r}(\bar{x},0) = \frac{r_{\mathrm{p}}^{\mathrm{max}}}{r_{\mathrm{H_2O}}}\,\gamma, \qquad 0 \le \bar{x} \le 1, \tag{S11}$$

$$\bar{c_2}(\bar{x},0) = 1, \qquad 0 < \bar{x} < 1, \tag{S12}$$

$$\bar{c_2}(0,0) = \frac{1-\bar{v}_1\bar{c_1}(0,0)}{\bar{v}_2}, \tag{S13}$$

$$\bar{\Gamma}(\bar{x},0) = \left(\Omega\arcsin\left(\left(\bar{r}(\bar{x},0)-1\right)^{-1}\right)\right)^{-1}, \qquad 0 < \bar{x} < 1, \tag{S14}$$

$$\bar{\beta} = \left(\bar{c_2}(\bar{x},0)\left[\frac{1}{\bar{\Gamma}(\bar{x},0)} - 1\right]\right)^{-1}, \qquad 0 < \bar{x} < 1, \tag{S15}$$

$$\text{BC - AI (bath)}: \quad \bar{c_1}(1,\bar{t}) = 0, \qquad \bar{t} > 0, \tag{S16}$$

$$\text{BC - H}_2\text{O (drop)}: \quad \bar{c_2}(0,\bar{t}) = \frac{1-\bar{v}_1\bar{c_1}(0,\bar{t})}{\bar{v}_2}, \qquad \bar{t} > 0, \tag{S17}$$

$$\text{BC - H}_2\text{O (bath)}: \quad \bar{c_2}(1,\bar{t}) = 1, \qquad \bar{t} > 0. \tag{S18}$$

$$\overline{V}_{\mathrm{Del}}(\bar{t}) = \frac{m_\infty\, c_{\mathrm{AI,0}}^{\mathrm{drop}}\, M_{\mathrm{w,AI}}\, \overline{V}(\bar{t})\, \bar{c}_1(0,\bar{t})}{\rho_{\mathrm{H_2O}}\, \bar{v}_2\, \bar{c}_2(0,\bar{t})}, \qquad \bar{t} > 0, \tag{S19}$$

$$\text{CCR mode}: \quad \frac{\mathrm{d}\theta}{\mathrm{d}\bar{t}} = \frac{-\Lambda(H)\ \left(1+\cos(\theta)\right)^2\ f(\theta)\, b^2}{r_{\mathrm{drop,0}}^2\, D_{\mathrm{AI}}^{\mathrm{bulk}}}, \qquad 0 < \bar{t} \le \bar{t}_{\mathrm{rec}}, \tag{S20}$$

$$\frac{\mathrm{d}\overline{V}}{\mathrm{d}\bar{t}} = \frac{-b^2\,\pi\,\Lambda(H)\, r_{\mathrm{drop,0}}\, f(\theta)}{D_{\mathrm{AI}}^{\mathrm{bulk}}\, V_0}, \qquad 0 < \bar{t} \le \bar{t}_{\mathrm{rec}}, \tag{S21}$$

$$\begin{aligned}\text{CCA mode}: \quad \frac{\mathrm{d}\overline{V}}{\mathrm{d}\bar{t}} = {} & \frac{-b^2\,\pi\,\Lambda(H)\, f(\theta_{\mathrm{rec}})}{D_{\mathrm{AI}}^{\mathrm{bulk}}\, V_0}\left(\frac{3\, g(\theta_{\mathrm{rec}})\, V_0\, \overline{V}}{\pi}\right)^{\frac{1}{3}} \\ & \times\left[\bar{\chi}\overline{V}\left(\frac{\overline{V}}{\overline{V}_{\mathrm{Del}}} - 1\right)\right]\left(1 - \frac{c_{\mathrm{AI,0}}^{\mathrm{drop}}}{c_{\mathrm{POD}(\Phi)}}\,\bar{c}_1\right) \\ & + \Lambda_2\, \overline{A}_{\mathrm{drop}}(\bar{t})\left[\overline{D}_2(\bar{x},\bar{t})\,\frac{\partial}{\partial\bar{x}}\left(\varepsilon(\bar{x},\bar{t})\bar{c_2}(\bar{x},\bar{t})\right)\right]\Bigg|_{\bar{x}=0}, \qquad H > \mathrm{POD_{shift}},\ \bar{t} > \bar{t}_{\mathrm{rec}},\end{aligned} \tag{S22}$$

$$\frac{\mathrm{d}\overline{V}}{\mathrm{d}\bar{t}} = \frac{-b^2\,\pi\,\Lambda(H)\,f(\theta_{\text{rec}})}{D_{\text{AI}}^{\text{bulk}}\,V_0}\left(\frac{3\,g(\theta_{\text{rec}})\,V_0\,\overline{V}}{\pi}\right)^{\frac{1}{3}}$$
$$+\,\Lambda_2\,\overline{A}_{\text{drop}}(\bar{t})\left[\overline{D}_2(\bar{x},\bar{t})\,\frac{\partial}{\partial\bar{x}}\left(\varepsilon(\bar{x},\bar{t})\overline{c_2}(\bar{x},\bar{t})\right)\right]\Bigg|_{\bar{x}=0}, \quad H \le \text{POD}_{\text{shift}},\ \bar{t} > \bar{t}_{\text{rec}}, \quad \text{(S23)}$$

$$\text{BCs - AI (drop)}:\ \frac{\mathrm{d}}{\mathrm{d}\bar{t}}\left[\overline{V}(\bar{t})\,\overline{c_1}(0,\bar{t})\right] = -\omega\overline{c_1}(0,\bar{t}) + \bar{\gamma}\overline{A}_{\text{drop}}(\bar{t})\left[\overline{D}_1(\bar{x},\bar{t})\frac{\partial}{\partial\bar{x}}\left(\varepsilon(\bar{x},\bar{t})\overline{c_1}(\bar{x},\bar{t})\right)\right]\Bigg|_{\bar{x}=0}, \quad \text{(S24)}$$

2 ISOLATED CUTICLE DATA

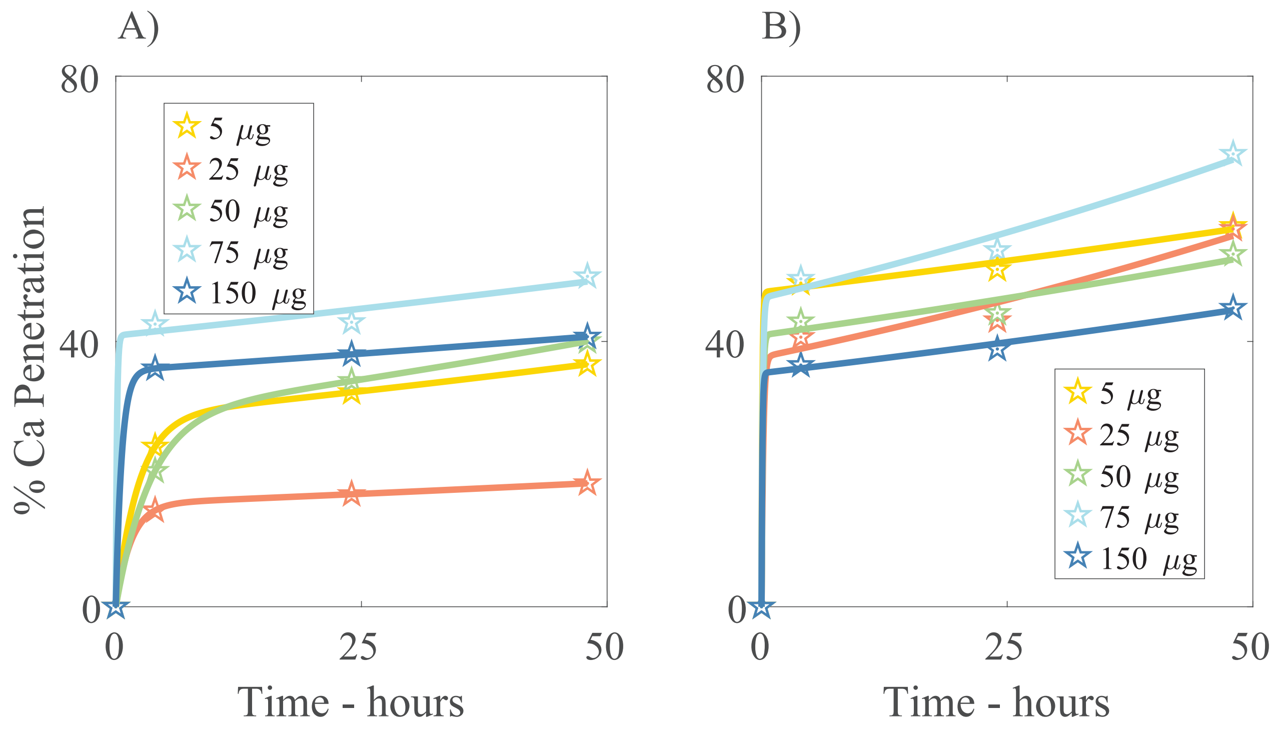

Figure S1: Influence of calcium (Ca) concentration on percentage Ca penetration. $CaCl_2$ is applied at 1, 5, 10, 15, and 30 g/L and each cuticle receives five 1μL drops totaling 5, 25, 50, 75 or 150 μg Ca per cuticle. $CaCl_2$ is shown on the left (A) and $CaCl_2$ with RSO 5 is shown on the right (B). Samples were taken 4, 24, and 48 hours after application at 70%RH and 20 °C. We have presented the Kraemer et al. (2009) data as a percentage and fitted with a linear combination of exponential functions in Equation (S25).

We have fitted both data sets in Figure S1 as a linear combination of exponential functions:

$$\%\ \text{Ca penetration}(t) = A\,\left[\mathrm{e}^{Bt} - \mathrm{e}^{Ct}\right], \quad \text{(S25)}$$

where % calcium (Ca) penetration is the mass percentage of Ca diffused through the cuticle as a function of time, t is time in hours and $A,\ B$ and C are the fitting coefficients. The data for $CaCl_2$ can be fitted with an R-squared value of 100%, with the exception of 75 μg, which has an R-squared value of 99.6%. The

data for $CaCl_2$ with RSO 5 can be fitted with a mean R-squared value of 99.7%. We note that this data has been fitted with an empirical equation here to merely aid in understanding of the data trends and this equation will not be utilized within the model. An example of the fitting parameters for 5 μg, for $CaCl_2$ with RSO 5 is:

$$\%\ \text{Ca penetration}(t) = 47.4\ \left[\mathrm{e}^{0.004\ t} - \mathrm{e}^{-12.3\ t}\right].$$

3 CONCENTRATION PROFILE RESULTS

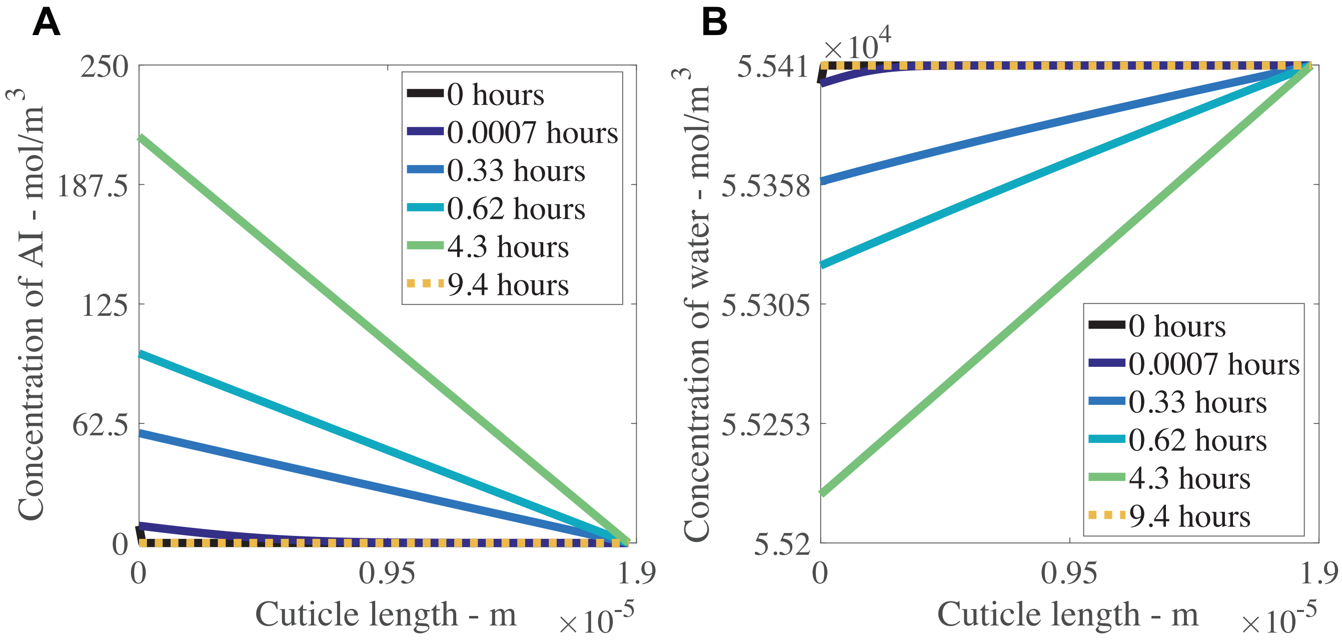

Figure S2: Plant cuticle model results for a single applied concentration of AI (A) and water (B), using parameters outlined in Table 2 with a single applied initial concentration of 1 g/L or 5 μg. The initial condition at $t = 0$ hours, is shown as a black line and the boundary conditions for the drop and bath are located at cuticle length $x = 0$ and $x = 1.87 \times 10^{-5}$ m respectively. The dashed yellow line on top of the black line is the final time at $t = 9.4$ hours, as shown in the legend.

4 POINT OF DELIQUESCENCE DATA

Table S1. POD data for the sensitivity analysis.

Humidity	ξ	Φ	POD
33%RH	27%RH	60%RH	5%RH
33%RH	-	-	62%RH
33%RH	-	-	97%RH
70%RH	27%RH	97%RH	5%RH
70%RH	−30%RH	40%RH	62%RH
70%RH	-	-	97%RH
90%RH	27%RH	97%RH	5%RH
90%RH	−30%RH	60%RH	62%RH
90%RH	-	-	97%RH

5 SENSITIVITY ANALYSIS

Table S2. Sensitivity rankings of parameters based on the one-factor-at-a-time method, calculated in 3 ways - percentage relative sensitivity at the final time (48 hours), S $_{\%}$, and the sensitivity based on the residual sum of squares, SS_{res} and root-mean-square error, RMSE over time, S $_{\text{SS}_{\text{res}}}$ and S $_{\text{RMSE}}$, as shown in Equations (S26 - S29).

Parameter	Description	S $_{\%}$	S $_{\text{SS}_{\text{res}}}$	S $_{\text{RMSE}}$
F_s	Fractal scaling - tortuosity	192%	13,237	73
r_p^{max}	Maximum aqueous pore radius	131%	9,210	50
H	Relative humidity	109%	6,532	39
η_{pore}	Aqueous pore density	99%	7,176	38
ξ	POD RH adjustment factor for RSO 5	84%	4,745	30
θ_0	Initial droplet contact angle	82%	5,051	31
k	Ion binding	76%	3,262	24
b	Cuticle thickness	72%	4,421	28
χ	Droplet evaporation scaling factor	69%	2,630	21
V_0	Initial droplet volume	63%	2,624	21
$c_{\text{AI},0}^{\text{drop}}$	Initial AI droplet concentration	10%	439	6

The sensitivities in Table S2 are a means of ranking the parameters, calculated in 3 ways. The first, percentage relative sensitivity at the final time (48 hours), S $_{\%}$, is found by comparing the ratio of the change in percentage penetration to the relative percentage change of the parameters that produce the highest and lowest penetration, P_{H} and P_{L}, at 48 hours, namely,

$$\text{S}_{\%} = \frac{\Delta\ \%\ \text{Penetration}}{\Delta\ \text{Parameter}}, \tag{S26}$$

$$\Delta\ \text{Parameter} = \frac{P_{\text{H}} - P_{\text{L}}}{P_{\text{H}}}. \tag{S27}$$

The second method of calculating the rankings is based on the residual sum of squares, SS_{res}. The partial effect value, S $_{\text{SS}_{\text{res}}}$ is found by comparing the average of the SS_{res} for the highest and lowest penetration, $\text{SS}_{\text{res, H}}$ and $\text{SS}_{\text{res, L}}$, with the penetration used for validation, $\text{SS}_{\text{res, valid}}$, compared to the relative percentage change of the parameter, Δ Parameter, namely,

$$\text{S}_{\text{SS}_{\text{res}}} = \frac{0.5\ (\text{SS}_{\text{res, H}} + \text{SS}_{\text{res, L}}) - \text{SS}_{\text{res, valid}}}{\Delta\ \text{Parameter}}. \tag{S28}$$

All values for SS_{res} are based on the experimental data, from 0 to 48 hours.

The third method is the same as Equation (S28), except is based on the root-mean-square error, RMSE, over time, namely,

$$\text{S}_{\text{RMSE}} = \frac{0.5\ (\text{RMSE}_{\text{H}} + \text{RMSE}_{\text{L}}) - \text{RMSE}_{\text{valid}}}{\Delta\ \text{Parameter}}. \tag{S29}$$

6 ION BINDING ANALYSIS

Table S3. Fitted parameter values for Figure 11 for fitting with and without ion binding. The root-mean-square error, RMSE, indicates the fit, where values closer to zero indicate improved fits.

F_s	k	η_{pore}	χ	RMSE
1.203	8.68e−16	2.18e15	0.04	5
1.203	0	2.18e15	0.04	32
1.3	0	2.18e15	0.04	31
1.138	3.74e−15	1.99e15	0.0023	8
1.3	0	1.99e15	0.0023	24
1.138	0	0.5e15	0.0023	31

REFERENCES

Kraemer T, Hunsche M, Noga G. Cuticular calcium penetration is directly related to the area covered by calcium within droplet spread area. *Scientia horticulturae* **120** (2009) 201–206. doi:10.1016/j.scienta.2008.10.015.
